# Virtue Discounting: Observability Reduces Moral Actors’ Perceived Virtue

**DOI:** 10.1162/opmi_a_00085

**Published:** 2023-07-26

**Authors:** Gordon T. Kraft-Todd, Max Kleiman-Weiner, Liane Young

**Affiliations:** Department of Psychology and Neuroscience, 140 Commonwealth Ave, Boston College, Chestnut Hill, MA 02467, USA; School of Engineering and Applied Sciences, Harvard University, 33 Kirkland St., Cambridge, MA 02138, USA

**Keywords:** virtue, observability, motivation, generosity, fairness

## Abstract

Performing prosociality in public presents a paradox: only by doing so can people demonstrate their virtue and also influence others through their example, yet observers may derogate actors’ behavior as mere “virtue signaling.” Here we investigate the role of *observability* of actors’ behavior as one reason that people engage in such “virtue discounting.” Further, we investigate observers’ motivational inferences as a mechanism of this effect, using the comparison of *generosity* and *fairness* as a case study among virtues. Across 14 studies (7 preregistered, total *N* = 9,360), we show that public actors are perceived as less virtuous than private actors, and that this effect is stronger for generosity compared to fairness (i.e., *differential virtue discounting*). Exploratory factor analysis suggests that three types of motives—principled, reputation-signaling, and norm-signaling—affect virtue discounting. Using structural equation modeling, we show that observability’s effect on actors’ trait virtue ratings is largely explained by inferences that actors have less principled motivations. Further, we leverage experimental evidence to provide stronger causal evidence of these effects. We discuss theoretical and practical implications of our findings, as well as future directions for research on the social perception of virtue.

## INTRODUCTION

*A [person] of the highest virtue does not keep to virtue and that is why [they have] virtue.*

*A [person] of the lowest virtue never strays from virtue and that is why [they are] without virtue.*

*—Lao Tzu, Tao Te Ching (Chapter 38, L.1-4; Tzu & Lau, 1963)*

Public prosociality presents a paradox: For an actor’s virtue to be known—and for their example to be followed—their morally good behavior must be observed by others; yet when an actor’s morally good behavior is observable, others may doubt the actor’s virtue. Resolving this paradox is particularly perplexing for early adopting advocates of uncommon prosocial behaviors (e.g., installing residential solar panels, buying electric vehicles, adopting vegan diets): can such an individual effectively demonstrate the moral worth of their behavior while avoiding observers’ skepticism of their moral character?

To illustrate this paradox, imagine browsing a printed list of people who donated the same amount to a charitable cause. The first two names listed are one of your friend’s and “Anonymous”; whose behavior is more indicative of their virtue? If you think your friend appears less virtuous by comparison, your intuitions are aligned with ancient philosophical arguments (Maimonides, 1170) borne out in recent empirical work (De Freitas et al., 2019). Still, your (less virtuous) friend is likely to have more prosocial influence on you than the (more virtuous) anonymous giver (Smith et al., 2015). From a consequentialist standpoint, your friend actually caused *more* overall good; why, then, do we think they’re *less* virtuous?

This paradox has long been puzzled over in the context of charitable giving (e.g., Lin-Healy & Small, 2012; Newman & Cain, 2014)—a prime example of behavior expressing the virtue of *generosity*—but are the dynamics the same when considering behaviors expressing other virtues (e.g., *fairness*)? This question is of theoretical interest for understanding social perception of prosociality, but it is also of practical interest for its downstream consequences: we can be more effective advocates if we can understand which types of behaviors face the paradox of public prosociality (and why).

### “Virtue Discounting” and Observability

We define a *virtue* as: a quality of individuals valued by their culture and expressed through a stable pattern of properly motivated behavior (Kraft-Todd et al., 2022). In the present work, we are particularly focused on how attribution of actors’ motivations is linked to perceptions of their virtue. Following most work in the Western philosophical and empirical traditions, many fundamental aspects of our concept of virtue can be traced to Aristotle’s *Ethics* (Aristotle, 1999). Namely, that virtue is trait-like, i.e., a stable, dispositional characteristic of individuals; that it is expressed through behaviors; and that it requires “proper” motivation (Cokelet & Fowers, 2019). Proper motivation evokes Aristotle’s concept of *eudaimonia* (often translated as “human flourishing”) which he meant as an end in itself (rather than a means to some other end; Aristotle, 1999), interpreted in recent work as, “one’s understanding of the virtue is itself motivating” (Cokelet & Fowers, 2019).

Moral judgments can range from assessments of specific behaviors to broad characterization of individuals, and people use the former as input to the latter (Hartman et al., 2022). Additionally, people often make judgments at the level of mental states and traits (Tamir & Thornton, 2018) as well as other “mental occurrents” (e.g., beliefs; Critcher et al., 2020)—when evaluating others’ moral characters from their behavior. Relevant to the present investigation, perceptions of actors’ motivations (Carlson et al., 2022) are a commonly documented type of mental state inference affecting such “person-centered” characterological moral judgments (Pizarro & Tannenbaum, 2012). To be clear, we differentiate judgments of *moral character* as domain-general assessments of individuals’ morality (e.g., on a spectrum from “good” to “bad”) from judgments of virtue as domain-specific assessments of individuals’ positive (moral) qualities.

In the attribution literature, there is a long history of work examining “discounting” (Kelley, 1972) of observers’ perceptions of the causes of actors’ behavior. This work has typically investigated different types of explanations (e.g., as attributable to the person vs. the situation) for actors’ behavior (McClure & Hilton, 1997). In the present investigation, we are focused on observers’ attributions of *motivations*, specifically, as explanations for actors’ behavior (Fein, 1996). In keeping with this work, we describe the phenomena in which observers’ motivational attributions lead them to believe that actors’ behavior is less attributable to actors’ virtue as “virtue discounting.”

Here we focus on the role of *observability*, i.e., the degree to which actors’ behavior is visible to uninvolved third parties (as opposed to recipients, interaction-partners, etc.), as a cause of virtue discounting. Observability is a central component of the theory of indirect reciprocity (Nowak & Sigmund, 1998), which explains how cooperation can evolve among unrelated individuals when there are rules (e.g., social norms) governing individuals’ behavior (Ohtsuki & Iwasa, 2006) and information regarding individuals’ (non-)adherence to these rules can be stored and transmitted (i.e., through their “reputations”). Implications of this theory are borne out in field experiments (for a review, see: Kraft-Todd et al., 2015) showing that making people’s behavior observable activates people’s reputational concerns, causing them to follow social norms supporting prosocial behavior. Despite the efficacy of observability for motivating such behavior (e.g., Yoeli et al., 2013), observers may be less likely to infer that the behavior it inspires is a signal of actors’ virtue because actors are not properly motivated. Thus, observers may infer (correctly) that actors are motivated by reputational benefit, rather than, e.g., genuine outrage at injustice, desire for environmental conservation, concern for others’ welfare, etc. In other words, observability introduces an ulterior motive (reputational concerns) that taint observers’ inferences of actors’ virtue because actors’ behavior is not (properly) motivated by pursuit of the virtue as an end in itself. Accordingly, we formulate our first preregistered hypothesis “virtue discounting”: *people will discount actors’ virtue when actors’ behavior is observable*.

### Generosity vs. Fairness as A Case Study in Virtue Discounting

Much existing empirical work demonstrates virtue discounting in the context of the virtue *generosity* (e.g., Newman & Cain, 2014). Previous work has argued that *fairness* is well-suited as a comparison virtue to generosity; for example, there is evidence that generosity and fairness differ across many psychological dimensions, as well as in the natural language people use to describe example behaviors of each virtue (Kraft-Todd et al., 2022). We build on much prior work distinguishing these virtues: in virtue ethics (e.g., as “natural” vs. “artificial” virtues; Hume, 1902); in recent theoretical arguments distinguishing these virtues’ functions (i.e., generosity enables individuals to make cooperative partnerships, whereas fairness enables individuals to avoid punishment from their cooperative partners; Shaw, 2016); and in empirical work demonstrating how these virtues can be operationally distinguished and shown to come into conflict (e.g., Kleiman-Weiner, Shaw, et al., 2017; Shaw & Olson, 2012). Taken together, fairness may serve as a valuable comparison to generosity for investigating virtue discounting across virtues.

As in previous work, we employ a “narrow” definition of both virtues. We define *generosity* as “giving an abundance of one’s money or time”, capturing most uses of the term, but not, e.g., generosity of attitudes (Gulliford & Roberts, 2018). We define *fairness* as “treating others equally and fairly, without bias”, capturing recent work on impartiality (Shaw, 2013), but not, e.g., work on fairness that focuses on the need of recipients (i.e., charity; Niemi & Young, 2017). Thus, our case study might be more precisely described as: “a case study of generosity vs. fairness (as impartiality).” To avoid confusion: we will henceforth use the terms *generosity* and *impartiality* when discussing our experiments—because these are the terms we use in our stimuli—but to refer to the respective virtues in keeping with the virtue literature, we will use the terms *generosity* and *fairness*.

Pertinent to the present investigation, there is also reason to believe that generosity and fairness might be discounted to different degrees. Fairness (as a component of *justice*) has long been considered enforceable through law whereas generosity has not (Schneewind, 1990). Consequently, there may be greater plausible deniability in attributing actors’ fairness (vs. generosity) to a desire to conform to formal regulations (and social expectations) rather than a desire to improve one’s reputation. It might be argued that behaving in accordance with social expectations is itself a means of improving one’s reputation (Ohtsuki & Iwasa, 2006). We posit, however, that such behavior will be seen as less self-serving than “morally motivated deviance” (Cramwinckel et al., 2015), e.g., when people engage in antisocial punishment, penalizing individuals who self-sacrifice to benefit the group because it goes against perceived norms (Herrmann et al., 2008). Consistent with this account, previous work distinguishing generosity and fairness provides evidence of the inverse; for example, in a resource distribution paradigm, adults (Shaw & Knobe, 2013) as well as children (Shaw & Olson, 2012) would rather throw away a windfall resource (the ungenerous but fair choice) in order to avoid appearing biased (i.e., rather than make the generous but unfair choice). Such decisions may be motivated by individuals’ perceptions that it would be worse to be seen violating a social expectation (to be unfair) rather than to merely be seen as ungenerous.

Further complicating the prosocial interpretation of generous behaviors, although generosity can be a signal of cooperative intent (i.e., willingness to provide benefits to others), it can also be a signal of wealth (Barclay, 2016), and thus individuals may show generosity so that others think they are rich. Also, the ideal of fairness (as impartiality) has a specific numerical connotation—i.e., to treat others with zero bias—whereas the ideal of generosity is effectively without ceiling (i.e., to be maximally generous, one can always give more). As a result, it is easier to coordinate around a (categorical) norm of fairness than a (continuous) norm of generosity. There is a wealth of evidence from mathematical models and empirical studies (Yoeli & Hoffman, 2022) suggesting that plausible deniability and categorical norms are two important mechanisms motivating human behavior and how it is perceived. In the present context, interpreting fairness compared to generous behaviors seems simpler, both because of the categorical nature of applicable norms, and also because of greater plausible deniability in the attribution of selfish motivations. Thus, we arrive at our second preregistered hypothesis, “differential virtue discounting”: *people will discount fairness (as impartiality) less than generosity*.

### Motivational Inference in Perceptions of Virtue

To this point, we have discussed “motivation” in a limited sense; i.e., as either the proper motivation requisite for virtue or as the motivation to benefit one’s reputation. Yet, prior work has explored many distinct motivations for prosocial behavior (Carlson et al., 2022; Kodipady et al., 2021; Narvaez & Snow, 2019; Reiss & Havercamp, 1998). To better understand the motives observers attribute to prosocial actors and how these contribute to virtue discounting, we focus on six motivations suggested by this literature: *self-presentation*, *norm-signaling*, *self-benefit*, *other-benefit*, *moral rules*, and *virtue identification*.

We opened with a paradox of public prosociality that highlights the tension between two motivations. Pulling in one direction (and to put a finer point on “reputational concerns”), we call the motivation to affect others’ impression of oneself *self-presentation*, following work documenting how people manage others’ impression of them through “self-presentation strategies” (Jones & Pittman, 1982). Pulling in the other direction, we call the motivation to lead by example *norm-signaling*, deriving from work on the role individuals have to influence social norms in a “grassroots” manner (Tankard & Paluck, 2016). Although the idea of “leading by example” is a topic of increasing theoretical (Henrich, 2009) and empirical (Kraft-Todd et al., 2018) interest, we are unaware of previous work investigating “leading by example” as a *motivation* (though see: Kodipady et al., 2021).

We briefly alluded to another pair of conflicting motivations in our discussion of observable generosity. A number of recent reviews have emphasized how fundamental the motivation of *other-benefit*, i.e., the desire to improve the welfare of others, is for motivating prosocial behavior (e.g., Keltner et al., 2014). The emphasis of these reviews stands in contrast to a historical bias, informed by the economic and evolutionary literatures (e.g., Friedman, 1953), to a reductive focus on the motivation of *self-benefit*, i.e., the desire to improve one’s own welfare.

Finally, we explore two identity-relevant motivations. Research on social norms has distinguished types of social rules guiding behavior, among which is the class of *moral rules* that individuals believe should guide behavior regardless of others’ expectations (i.e., as opposed to “socially-dependent” social rules that rely on others’ expectations, moral rules are “socially-independent”; Bicchieri, 2006; Levine et al., 2020). This motivation interestingly differs from the previously discussed motivations because it is not outcome-oriented; instead, it derives from an individual’s socially-independent moral values. Another socially-independent motivation for virtuous behavior derives from actors’ sense of identity, as explored in research on, e.g., “moral identity” (Aquino & Reed, 2002), “moral consistency” (Kleiman-Weiner, Saxe, et al., 2017; Mullen & Monin, 2016), and “self-concept maintenance” (Mazar et al., 2008). In the context of the present work, we study whether individuals’ identification with the specific virtue under investigation might be a motivation for virtuous behavior, and call this *virtue identification*.^1^ This motivation most closely approximates “proper” motivation in our definition of virtue.

In keeping with previous work, our third preregistered hypothesis is: *(differential) virtue discounting will be explained by observers’ inferences that public actors have more selfish motivations than private actors*. In the present work, however, we extend this previous work which typically invokes “selfish” motivations (generally) and reputational concerns (specifically) as drivers of virtue discounting (e.g., Raihani & Power, 2021). We do not intend our investigation to be an exhaustive account of all possible motivational inferences, but our preregistered exploratory factor analyses and structural equation models shed new light on the social perceptions underlying virtue discounting.

## GENERAL METHODS

We describe the general procedure in common across experiments here, and summarize more fine-grained detail in Table 1 (as well as Supplement Section 1). In the Supplement, we provide justifications for changes in experiment design across experiments (Section 2), and also provide complete experimental instructions (Section 8). All measures, manipulations, and exclusions in all experiments are disclosed across the main text and Supplement. All experiments were conducted online using Qualtrics survey software. A convenience sample of participants were recruited using the crowdsourcing tool Cloud Research and Amazon Mechanical Turk (“mTurk”; Arechar et al., 2017; Berinsky et al., 2012). We excluded duplicate Amazon worker IDs and IP addresses to prevent analyzing multiple observations per participant (as well as participants who dropped out prior to assignment to condition), yielding a final sample of *N* = 9,360 participants (51.2% female, *average age* = 37.7 years). Informed consent was obtained from all participants, who completed experiments in *mean* = 5 minutes and were paid *mean* = $0.89 for their participation. For conciseness, we abbreviate references to specific experiments as “E#” (e.g., E3 = Experiment 3).

**Table 1.** Key elements of stimuli. Shown are characteristics and language of key elements of stimuli bearing on hypotheses in all experiments (for complete experimental instructions, see Supplement Section 8).

**Exp. num.**	**N**	**Stimuli information**		**Stimuli language**			
				**Virtue (example behaviors)**		**Observability**	
		**Motive stipulated?**	**Example behaviors**	**Generosity**	**Impartiality**	**Private**	**Public**
1	389	Yes	(none)	[Virtue definition]: "Generosity usually means giving an abundance of one’s money or time"	[Virtue definition]: Impartiality usually means treating everyone equally and fairly, without bias	Though she is [G/I] when she is with others, she is especially [G/I] when no one is watching since she knows that acting in this way is consistent with her values.	She is especially [G/I] when others are watching her act since she knows that her reputation for being [G/I] will improve.
2	394						
3	394						
4	388		Experimenter-generated	- volunteered at a homeless shelter - donated money to charities like Doctors without Borders - donated blood during a blood drive	- made sure everyone at a social gathering receives the same amount of food - divided work evenly among all participants in a group project - made auditions or job applications blind so that subtle, unconscious biases against particular genders or ethnicities don’t enter into the decision-making process		
5	393					Though she is [G/I] when she is with others, she is even [G/I] when no one is watching.	She is especially [G/I] when others are watching her act.
6	393		Participant-generated	- bought a friend an expensive gift - gave a waiter a large tip - stayed late to help a coworker	- gave her children equal allowances - conducted a blind audition - drew names from a hat for a project at work		
7	582	No				She did these things in private; therefore, other people did not know that she did them.	She did these things in public; therefore, other people knew that she did them.
8	577						
9	386						
10	663	Yes*					
11	394	No		- gave someone a hand carrying groceries - shared food with friends - gave someone praise	- stayed out of an argument - divided food by cutting and letting the other person pick which piece they want - helped to moderate when your friends had a disagreement		
12	377						
13	1,770			- bought someone a meal - donated blood - volunteered at an animal shelter	- divided food by cutting it and letting the other person pick which piece they wanted - learned to pronounce others’ names regardless of their country of origin - listened to both parties in a conflict equally		
14	2,260	Yes*					

After providing consent and entering their mTurk ID, we randomly assigned participants to one between-subjects condition. We crossed our primary manipulations in all experiments in a 2 (virtue: generosity vs. impartiality) × 2 (observability: public vs. private) factorial design. First, participants read text delivering our virtue manipulation, adapted from Merriam-Webster.com (see Table 1). We asked participants to imagine that they know someone (henceforth: “the actor”) whom we named using a list of common female names in the US (because we were not interested in the effect of actor gender on the dependent variables, we used all female names). We then told participants that the actor engaged in a set of three behaviors demonstrating [generosity/impartiality]. Then, participants read text delivering our observability manipulation (see Table 1, Columns 7 and 8).

The behaviors used as stimuli were generated in a rigorous “bottom-up” manner (Kraft-Todd et al., 2022). First, participants were randomly assigned to virtue condition (generosity vs. impartiality) and asked them to provide behaviors that demonstrated the virtue using free-response text. Second, an independent sample (also randomly assigned to virtue condition) was recruited to rate a subset of these participant-generated behaviors on nine underlying dimensions impacting moral judgment. Finally, to avoid idiosyncrasies of any specific behavior, a set of three behaviors were selected that described hypothetical actors engaging in to create a general impression of the actors as demonstrating each virtue in participants’ minds. Previous work found no significant differences in participants’ ratings between the sets of behaviors we used as stimuli in the generosity and impartiality conditions of each experiment across three dimensions: moral goodness, descriptive normativity, and the extent to which behavior is indicative of the actor’s consistency across situations (Kraft-Todd et al., 2022). This evidence speaks against alternative explanations of our effects, e.g., based on the diagnosticity of the behavior (Mende-Siedlecki et al., 2013) for actors’ virtue.

Following the stimuli, we presented participants with the two primary dependent measures and six secondary dependent measures (see Table 2) in randomized order. All dependent measures were answered on 100-point unmarked slider scales with extreme anchors labeled (and midpoints labeled for primary dependent measures). At the end of each experiment, we presented participants with basic demographic questions in randomized order (see Supplement Table 2 for summary of participant demographics by experiment).

**Table 2.** Dependent measures. Shown are labels (Column 2) and item wording (Column 3) for dependent measures used in all experiments.

	**Item**	**Wording**
Primary dependent measures	Moral goodness	“How morally good is [the actor]?”
	Trait ratings	“How [generous/impartial] is [the actor]?”
Secondary dependent measures (motivational inferences)	Moral rule	“… because she thinks it is the right thing to do?”*
	Virtue identification	“… because she wants to be [generous/impartial]?”*
	Other-benefit	“… because she wants to benefit others?”*
	Self-presentation	“… because she is trying to make others think she is [generous/impartial]?”*
	Self-benefit	“… because she thinks she will personally benefit from acting this way?”*
	Norm-signaling	“… because she wants others to be [generous/impartial], and she is trying to lead by example?”*

* Preceded by: “How much do you think [name] is motivated to act [generously/impartially]…”

We conducted analyses using STATA (16.1) and R software (4.1.2). We obtained effect sizes (Cohen’s D) through use of an online calculator (Lenhard & Lenhard, 2016). For regression analyses, we compute pairwise comparisons of estimated marginal cell means corrected for multiple comparisons using Scheffe’s adjustment (Winer et al., 1991; though results are equivalent using Bonferroni correction). Structural equation models were constructed using standardized variables (Hayes, 2013), and indirect effects are calculated using the multivariate delta method (Sobel, 1982) with bootstrapped standard errors (UCLA: Statistical Consulting Group, 2021). Prior to conducting E8, we conducted a power analysis using the Superpower package in R (Lakens & Caldwell, 2021), using data from E7. With a desired effect size of *d* = .30 (for the virtue * observability interaction; see Supplement Section 3 for effects by experiment), our sample size of *N* = 100 per cell was powered at 83.25% with an alpha level of .05. For E1–7, we used the heuristic of recruiting *N* = 100 per cell. Sample size for each experiment was determined before any data analysis. Sensitivity analyses were conducted using G*Power (3.1.9.7) software (Faul et al., 2007).

Below we present these preregistered analyses using data aggregated across all of our experiments (though we also provide precise preregistered analyses in the Supplement (see Supplementary Tables 4–7). Although our preregistered hypotheses include both of our primary dependent measures (i.e., moral goodness ratings and trait ratings), these measures were highly correlated (across all experiments: *r* = .74, *p *< .001), so we present trait rating results here for conciseness. Results do not qualitatively differ if moral goodness ratings are used instead (see Supplementary Tables 5 and 7). E10, E11, E13, and E14 included preregistered exclusions for participants who failed attention checks (average failure rate across studies = 12.2%). To maintain consistency in our results across experiments and to be maximally inclusive of data we collected, the results presented below include participants who failed attention checks (*N* = 720, 7.7% of total *N*). Results are robust to excluding these participants (Supplementary Table 8).

Preregistrations were conducted for: E8 (https://aspredicted.org/FLG_VRD), E9 (https://aspredicted.org/NUP_ESB), E10 (https://aspredicted.org/FCO_HXM), E11 (https://aspredicted.org/2Y2_YKT), E12 (https://aspredicted.org/AES_HGO), E13 (https://aspredicted.org/G6S_S35), and E14 (https://aspredicted.org/SHW_9PN). All data and code are publicly available at: https://osf.io/sud3m/?view_only=380a169770b9474f93d2b5b73adc7410.

## ANALYSIS 1. VIRTUE DISCOUNTING AFFECTS GENEROSITY MORE THAN IMPARTIALITY

The purpose of Analysis 1 is to test for evidence of our first two hypotheses (preregistered in E8-14): 1) virtue discounting: people will discount actors’ virtue when actors’ behavior is observable; and 2) differential virtue discounting: people will discount fairness (as impartiality) less than generosity. To do so, we examine participants’ ratings of actors’ trait virtue in our hypothetical vignettes across conditions, using multivariate regression (collapsing across experiments) as well as meta-analysis (using the “metan” package in R; Olivoto & Lúcio, 2020).

We also provide an exploratory test of the directionality implied by our *virtue discounting* terminology, i.e., that participants discount public virtue rather than reward private virtue. In two experiments (E7 and E8, see Supplement Section 4) we added an additional “baseline” observability condition that provided no information about observability (i.e., we simply omitted the line, “She did these things in [public/private]; therefore, other people [knew/did not know] that she did them” from our experimental instructions; see Supplement Section 8). Crucially, we note that the example behaviors used as stimuli in these experiments were not rated differently on the dimension of “potential for anonymity” in our previous data (Kraft-Todd et al., 2022), supporting our interpretation that participants did not perceive differences across virtues in the extent to which these behaviors were known to uninvolved third-parties. By comparing participants’ trait ratings in the public and private observability conditions to the baseline observability condition, we provide a test of whether public virtue is discounted, or instead, whether private virtue is rewarded.

### Methods

We use data from all conditions in all experiments in which we manipulated observability (*N* = 8,969; 51.2% female, average age = 37.8 years). First, we conduct a multivariate regression analysis predicting moral goodness and trait ratings by our virtue manipulation (generosity vs. impartiality), observability manipulation (public vs. private), and their interaction, with Experiment as a covariate. Second, we conduct a random-effects meta-analysis on the effect sizes from the virtue * observability interaction.^2^

### Results

As predicted in our first preregistered hypothesis (*virtue discounting*), we find a significant effect of our observability manipulation, collapsed across virtue condition, on trait ratings (*F*(1, 8954) = 545.86, *p* < .001, *d* = .49) such that public actors (*m* = 72.06, 95% CI [71.41, 72.71]) are perceived as significantly less virtuous than private actors (*m* = 82.30, 95% CI [81.71, 82.88]). Sensitivity analysis revealed that (at *p* = .05 and power = 80%) the minimum detectable effect size for this test is *d* = .06.

**Figure 1.**
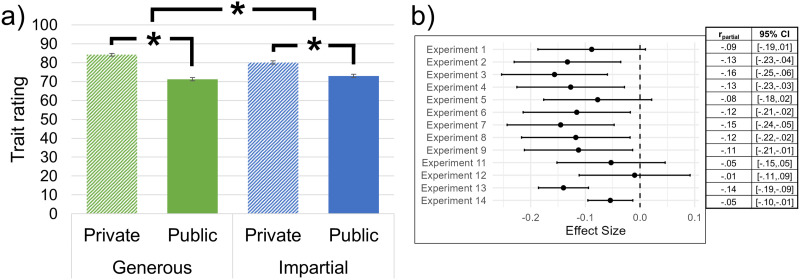
**Virtue discounting (i.e., public actors are rated less virtuous than private actors) affects generosity more than impartiality.** Shown are **a)** means (with 95% CIs) of trait ratings (0–100 unmarked slider) collapsed across all 14 experiments, as a function of whether the actor is said to have engaged in generous (green) or impartial (blue) behaviors and whether the actor is said to have engaged in behaviors publicly (solid) or privately (lines). Significant contrasts denoted with (*). From left to right: private generosity *N* = 2,397, public generosity *N* = 2,430, private impartiality *N* = 2,082, public impartiality *N* = 2,071. **b)** Random effects meta-analysis of the virtue * observability interaction on trait ratings across 13 experiments (E10 only investigated generosity), *N* = 8,317. Effect sizes are shown as partial correlation coefficients; error bars indicate 95% CIs. The relative sizes of the boxes indicate the weighting assigned to the experiments by the meta-analysis.

As predicted in our second preregistered hypothesis (*differential virtue discounting*), we also find a significant interaction between virtue and observability on trait ratings (*F*(1, 8952) = 42.18, *p* < .001, *d* = .14; see Figure 1). Sensitivity analysis revealed that (at *p* = .05 and power = 80%) the minimum detectable effect size for this test is *d* = .06. Publicly generous actors are perceived as significantly less virtuous (*m* = 71.26, 95% CI [70.35, 72.17]) than privately generous actors (*m* = 84.20, 95% CI [83.43, 84.96], Scheffe’s *t* = −21.62, *p* < .001, *d* = .61). Publicly impartial actors (*m* = 72.99, 95% CI [72.07, 73.92]) are also perceived as significantly less virtuous than privately impartial actors (*m* = 80.10, 95% CI [79.23, 80.98], Scheffe’s *t* = −11.18, *p* < .001, *d* = .34). Providing the crucial test of our preregistered differential virtue discounting hypothesis, a Wald test (*χ*^2^(1) = 42.70, *p* < .001) reveals that the effect of observability is greater for generosity (*contrast* = −12.91, 95% CI [−14.08, −11.75]) than impartiality (*contrast* = −7.19, 95% CI [−8.45, −5.92]).

We also provide multiple demonstrations of the robustness of our effects and our interpretation. A random-effects meta-analysis on the 13 virtue * observability interaction effect sizes reveals that generosity is discounted to a greater extent than impartiality (*r*_*partial*_ = −.10, 95% CI [−.13, −.07], *Z* = 7.50, *p* < .001; see Figure 1b). Further, we do not observe evidence of heterogeneity in effect size across experiments (*χ*^2^(12) = 14.93, *p* = .245), implying that this result is robust to differences in experimental design. Note that across all experiments, the virtue * observability interaction is either significant, such that generosity is discounted more than impartiality, or it is not significant; it is never the case that impartiality is discounted more than generosity. We further demonstrate the robustness of our effects by replicating these results in nearly all meaningful subsets of our experiments; importantly, including among our preregistered experiments (see Supplement Section 3).

Finally, we provide evidence in support of the directionality implied by our *virtue discounting* terminology. Comparing participants’ trait ratings in the public and private observability conditions to the baseline observability condition (see Supplement Section 4), we find that, compared to generous actors described without observability information (i.e., the baseline condition), privately generous actors are perceived as equivalently virtuous (*contrast* = −3.17, 95% CI [−8.65, 2.31], Scheffe’s *t* = −1.93, *p* = .571), whereas publicly generous actors are perceived as significantly less virtuous (*contrast* = −6.23, 95% CI [−11.71, −.75], Scheffe’s *t* = −3.79, *p* = .014). We interpret this result as showing that, consistent with our terminology, observers discount public virtue. In these experiments, impartial actors’ virtue was not discounted (comparing public to private observability conditions: *contrast* = −.43, 95% CI [−5.95, 5.09], Scheffe’s *t* = −.26, *p* = 1.000), so this comparison cannot be made for impartiality. Still, these results are consistent with one motivation for our *differential virtue discounting* hypothesis: although both conformity with social norms (Ohtsuki & Iwasa, 2006) and “morally motivated deviance” (Cramwinckel et al., 2015) may be means by which to enhance one’s reputation, our baseline condition results are consistent with the idea that perceived reputation management (through publicly observable virtuous behavior) may negatively impact observers’ perceptions of actors’ virtue more strongly in the case of the latter (here, generosity) compared to the former (here, impartiality).

### Discussion

Here we provide robust evidence in support of both of our first two preregistered hypotheses. First, our results demonstrate *virtue discounting*; participants rated public actors as less virtuous than private actors. Second, our results demonstrate *differential virtue discounting*; participants discounted public (compared to private) generosity to a greater extent than they discounted public (compared to private) impartiality. It is worth noting that, in both analyses, although observability reduces participants’ ratings of actors’ trait virtue, public actors are still qualitatively perceived as virtuous (i.e., mean ratings are above the response scale midpoint). These results are consistent with much previous work showing virtue discounting of generosity (e.g., Lin-Healy & Small, 2012). Further, it is the first demonstration, to our knowledge, of virtue discounting of impartiality. Finally, we provide evidence in support of the directionality implied by our *virtue discounting* terminology. Namely, participants rate publicly generous actors as less virtuous than privately generous actors and also generous actors described without observability information (i.e., the baseline condition), but they do not rate the latter two types of actors differently. This result implies that *public virtue is discounted* (although without this evidence it might have been argued that *private virtue is rewarded*). Next, we turn to our analyses showing motivational inferences as a mechanism of observability in virtue discounting.

## ANALYSIS 2. VIRTUE DISCOUNTING DUE TO OBSERVABILITY CAN BE EXPLAINED BY MOTIVATIONAL INFERENCES

The purpose of Analysis 2 is to explore the role of motivational inferences as a mechanism of observability in virtue discounting, testing our third preregistered hypothesis (in E8, E9, and E11–13) that *virtue discounting will be explained by observers’ inferences that public actors have more selfish motivations than private actors*. It would follow from our previous results to explore how motivational inferences mediate the virtue * observability interaction, i.e., accounting for the *differential virtue discounting* effect (this analysis presented in Supplement Section 5). An important result of that analysis, however, is that there are not qualitative differences in the motivational inferences driving virtue discounting of generosity compared to impartiality (i.e., it is not the case that different types of motivations explain virtue discounting for each virtue, only the degree to which observers infer these motivations). We acknowledge that this may be due to the fact that our exploration of motivational inferences was not exhaustive (and comment on this issue in greater detail in the General Discussion). That is, a similar investigation employing a broader range of motivational inferences might find that there *are* distinct motivational inferences driving virtue discounting in generosity compared to impartiality. Though we admit this possibility, here we present an analysis of how motivational inferences explain *virtue discounting*, i.e., collapsing across generosity and impartiality.

Additionally, we believe that our investigation of the mechanism of observability in virtue discounting could be of greater theoretical interest (i.e., than such an analysis of differential virtue discounting) because it could suggest a generalizable mechanism explaining virtue discounting via motivational inferences across an even broader range of virtues. Since we only use two virtues as stimuli, further research will be needed before such conclusions could be drawn. Still, we believe our analysis is an important first step towards such work.

### Methods

We use data from all experiments in which we measured motivational inferences (E6–9 and E11–13; *N* = 4,087; 49.9% female, average age = 37.9 years). We present correlations among these items in Supplement Section 6. Here we present an exploratory factor analysis (EFA; preregistered in E8, E9, and E11–13) of motivational inference items to better understand their latent structure, and then fit a multiple mediation model using these factor scores. We constructed generalized structural equation models to compute the mediation results (see General Methods for more details), and present alternative model specifications in Supplement Section 7.

### Results

We begin with an EFA (preregistered in E8, E9, and E11–13) of our six motivational inference items using iterated principal factors and oblique rotation. The analysis yielded two factors explaining 95.5% of the variance. Factor 1 explained 61.0% of the variance, and items with high loadings (> .6) were: *moral rule*, *virtue identification*, and *other-benefit*. Following our previous work (Kraft-Todd et al., 2022), we labeled Factor 1 “principled” because these motivations pertain either to actors’ moral beliefs/identity (i.e., moral rule and virtue identification) or prosociality (other-benefit). Factor 2 explained 34.4% of the variance, and we labeled it “reputation-signaling” due to high loadings (> .6) by the items: *reputational benefit* and *self-benefit*.

We note, however, that our norm-signaling item loads almost equivalently on these factors (*principled* = .39; *reputation-signaling* = .45). Because norm-signaling did not load uniquely on either factor, and also because we are specifically interested in this novel construct, we therefore conduct a second EFA omitting this item, with the intention to use the resulting factor scores in addition to norm-signaling ratings as mediators in our subsequent analysis. This second EFA (also using iterated principal factors and oblique rotation) retains the same two factors, on which all items load as described above. We observe that they also explain similar proportions of the variance among motivational inference items (*principled* = 69.2%; *reputation-signaling* = 28.0%), and that these factors are moderately and negatively correlated (*r* = −.46).

**Figure 2.**
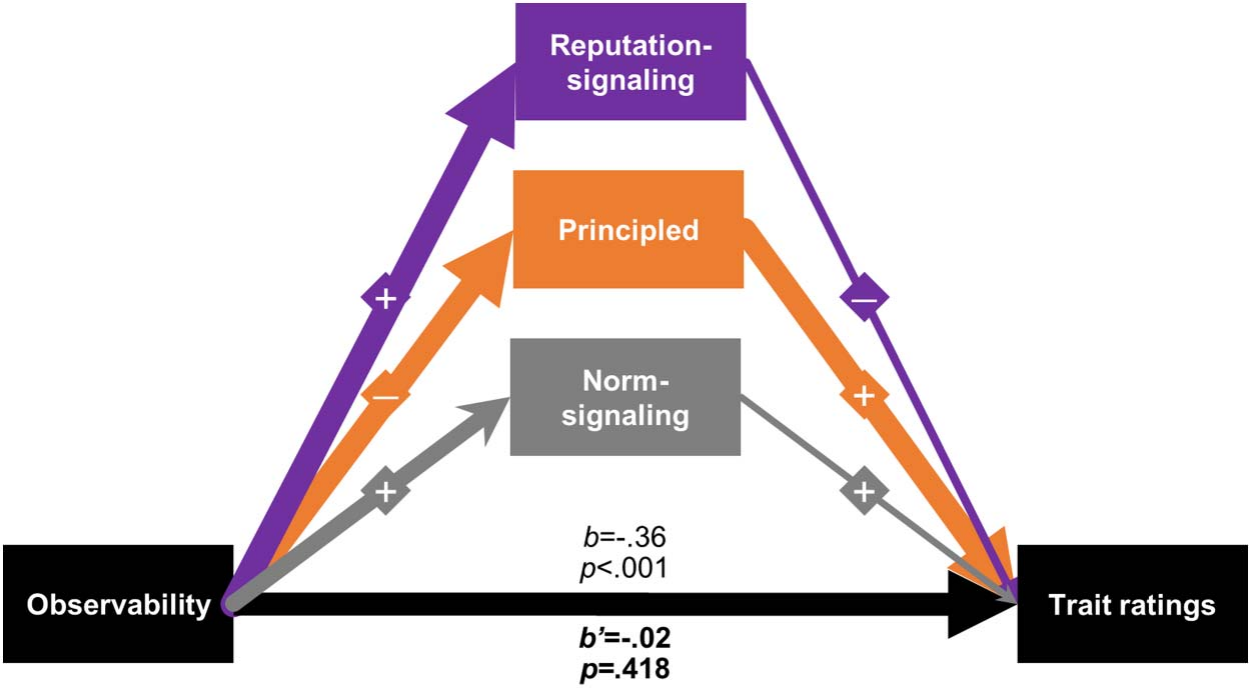
**The effect of observability on virtue discounting is explained by inferences that actors have lower principled motivations.** Shown is a generalized structural equation model (showing multiple mediation) of the effect of observability on trait ratings by motivational inference factor scores and norm-signaling item collapsed across virtue (*N* = 4,087). Bottom arrow (black) represents the effect of the observability manipulation (public compared to private condition) on trait ratings with (b) and without (b’) mediators as covariates. From left-to-right, the first set of arrows represents the effect of the observability manipulation on mediators, and the second set of arrows represents the correlation of mediators with trait ratings. Line thickness represents correlation strength; “+” and “−” represent correlation direction; all variables standardized for this analysis.

Next, we examine the mediation of observability on trait ratings by the two motivational inference factor scores plus the norm-signaling item (collapsed across virtue, controlling for Experiment and the covariance among mediators). Participants infer that, compared to private actors, public actors have significantly lower *principled* motivation (*b* = −.56, 95% CI [−.62, −.50], *p* < .001; see Figure 2), and significantly higher *reputation-signaling* motivation (*b* = .67, 95% CI [.62, .73], *p* < .001) as well as *norm-signaling* motivations (*b* = .28, 95% CI [.22, .34], *p* < .001). Next, we find that participants’ trait ratings are significantly associated with their motivational inferences, such that higher *principled* (*b* = .55, 95% CI [.53, .58], *p* < .001) and *norm-signaling* (*b* = .05, 95% CI [.03, .07], *p* < .001) inferences are associated with higher trait ratings, while higher *reputation-signaling* inferences are associated with lower trait ratings (*b* = −.07, 95% CI [−.09, −.04], *p* < .001).

Finally, we turn to the mediation results. Restating the *virtue discounting* result we present in Analysis 1, the total effect of observability on trait ratings is significant, such that participants rate public actors as less virtuous than private actors (*b* = −.36, 95% CI [−.41, −.31], *p* < .001). The direct effect of observability on trait ratings (i.e., accounting for indirect effects through motivational inferences) is not significant (*b’* = −.02, 95% CI [−.05, .02], *p* = .418), implying full mediation (95.6% of the total effect). Calculating indirect effects as percent of total effect, *principled* motivation accounts for 86.6% of this mediation, *reputation-signaling* motivation accounts for 12.7%, and *norm-signaling* motivation accounts for −3.7%.

### Discussion

Here we provide evidence consistent with an explanation of observability’s effect on virtue discounting through motivational inferences. In short, participants’ motivational inferences (i.e., *reputation-signaling* factor score, *principled* motivation factor score, and *norm-signaling* item) mediate 95.6% of the effect of our observability manipulation (i.e., public vs. private conditions) on trait ratings (collapsed across virtue condition). Although our *reputation-signaling* and *principled* motivational inference factors intuitively seem like conceptual opposites, we note that they are interestingly only moderately negatively correlated (*r* = −.46). Further, our finding that the *norm-signaling* motivational inference item does not uniquely load on either of our two other motivational inference factors suggests that this construct may have a unique role in contributing to an explanation of virtue discounting (Kodipady et al., 2021).

The results of this analysis only weakly support our third preregistered hypothesis (i.e., that virtue discounting will be explained by observers’ inferences that public actors have more selfish motivations than private actors). Despite the finding that an increase in (“selfish”) *reputation-signaling* motivational inferences account for 12.7% of the virtue discounting effect, 86.6% of the effect was explained through a decrease in *principled* motivational inferences. Our mediation result is robust to alternative model specifications, including one in which we model the mediating effect of the *reputation-signaling* factor score through the *principled* motivation factor score (see Supplement Section 7). That is, it might have been the case that participants perceived publicly virtuous actors to have greater *reputation-signaling* motivation, and therefore participants perceived them to have lower *principled* motivation, but our alternative model specification does not support such an account. This finding stands somewhat at odds with previous work showing that selfish motivations account for virtue discounting (e.g., Newman & Cain, 2014), although it should be noted that previous work did not simultaneously test multiple motivations that might explain this effect.

## ANALYSIS 3. OBSERVABILITY DOES NOT CAUSE VIRTUE DISCOUNTING WHEN OBSERVERS KNOW ACTORS’ MOTIVATIONS

The purpose of Analysis 3 is to provide stronger causal evidence that motivational inferences explain why observability causes virtue discounting. Our results from Analysis 2 suggest that observability engenders ambiguity in observers’ motivational inferences; i.e., observers can attribute different—and even conflicting—motivations to publicly virtuous actors. To reiterate, participants inferred that, compared to private actors, public actors had lower *principled* motivation and higher *reputation-signaling* motivation (both associated with participants rating actors as less virtuous). Yet, participants also inferred that public actors had higher *norm-signaling* motivation, which is associated with participants rating actors as *more* virtuous.

Rather than rely on correlational mediation (as in Analysis 2), here we leverage a design feature of many of our experiments (E1-6, and some conditions in E10 and E14) in which we explicitly manipulated actors’ motivation alongside observability. By comparing the effect of our observability manipulation among conditions in which we stipulate actors’ motivation to those in which we *do not* (as in E7-9, E11-13, and other conditions in E10 and E14), we can test the “motivational ambiguity hypothesis” (preregistered in E10 and E14): *the main effect of observability (i.e., public vs. private) on ratings of trait virtue will be substantially reduced when we stipulate actors’ motivation compared to when we do not*. To put this hypothesis plainly: observers may discount virtue because they are uncertain about actors’ motivations (as in our “no motive” conditions, and presumably in real life). Explicitly providing people with information about actors’ motivations, therefore, should drastically reduce the effect of observability on perceptions of actors’ virtue.

### Methods

We use data from all conditions in all experiments in which we manipulated observability (same as Analysis 1; *N* = 8,969; 51.2% female, average age = 37.8 years), subgrouping by manipulations of actors’ motivation in our hypothetical vignettes (*principled*, *N* = 2,151 vs. *reputation-signaling*, *N* = 2,150 vs. no motivation stipulated, *N* = 4,671). Although details varied by experiment (see Table 1), our manipulations of actor motive generally stipulated that actors either simultaneously had *principled* but not *reputation-signaling* motivations or *reputation-signaling* but not *principled* motivations. For conditions in which we did not stipulate actor motivation, we simply omitted language regarding actors’ motivation.

As in Analysis 2, here we are primarily interested to explore the observability * motive interaction collapsed across virtues; we therefore include the virtue manipulation as a covariate in the results presented here (see Supplement Section 3 for results disaggregated by virtue as well as a demonstration that these results are robust to simply omitting the virtue manipulation from the model presented below). As a result, the regression analysis presented here predicts moral goodness and trait ratings by the interaction of the observability manipulation (public vs. private) and the actor motive manipulation (reputation-signaling vs. principled vs. none), with Experiment and the virtue manipulation as covariates.

### Results

**Figure 3.**
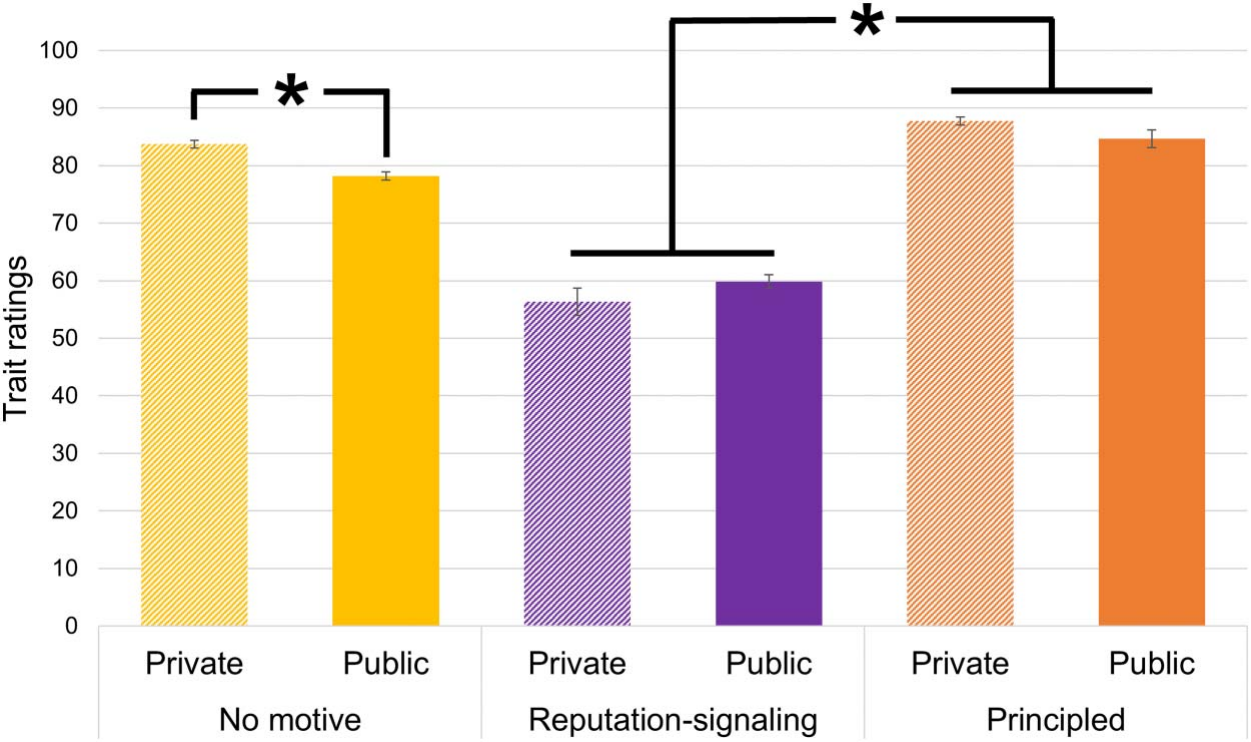
**Observability does not affect actors’ perceived virtue when their motivation is stipulated.** Shown are means (with 95% CIs) of trait ratings (0–100 unmarked slider), as a function of whether the actor is said to have engaged in behaviors privately (lines) or publicly (solid) and whether the actor’s motivation is stipulated as: none (yellow), reputation-signaling (purple), principled (orange). Significant contrasts denoted with (*). From left to right: private no motive *N* = 2,312; public no motive *N* = 2,360; private reputation-signaling *N* = 486; public reputation-signaling *N* = 1,669; private principled *N* = 1,681; public principled *N* = 472.

Overall, we find a significant interaction between observability and motive on trait ratings (*F*(2, 8960) = 26.36, *p* < .001, *d* = .15). Sensitivity analysis revealed that (at *p* = .05 and power = 80%) the minimum detectable effect size for this test is *d* = .07. When we do not stipulate actor motivation, we again observe *virtue discounting*; i.e., public actors are rated as less virtuous than private actors (*contrast* = −5.60, 95% CI [−7.41, −3.78], *t* = −10.27, *p* < .001, see Figure 3).

Consistent with our hypothesis (preregistered in E10 and E14), we observe that public and private actors are not rated differently on trait virtue when we stipulate that actors have *reputation-signaling* motivation (*contrast* = −2.59, 95% CI [−6.19, 1.01], *t* = −2.39, *p* = .334) or *principled* motivation (*contrast* = 2.80, 95% CI [−.82, 6.43], *t* = 2.58, *p* = .249).

### Discussion

In this analysis, we show that observability does not affect participants’ ratings of actors’ trait virtue when we stipulate actors’ motivation. Specifically, when we describe actors as having *principled* (but not *reputation-signaling*) motivations or *reputation-signaling* (but not *principled*) motivations, participants’ trait ratings do not differ by observability condition (i.e., public vs. private). We note that, qualitatively, participants rate actors’ trait virtue slightly positively (i.e., close to, but above the response scale midpoint) when we stipulate that actors have reputation-signaling motivations. Comparing these results to those of Analysis 1, it is also interesting to note that the magnitude that participants discount actors’ trait virtue is roughly twice as great when we explicitly stipulate actors’ reputation-signaling motivation (as we do here) compared to when we merely describe actors’ behavior as publicly observable (as in Analysis 1).

This analysis provides stronger causal evidence of the account we propose in Analysis 2: information about actors’ motivations impacts observers’ ratings of actors’ trait virtue. When this information is not explicit—as in the design of Experiments investigated in Analysis 2 and presumably in real life—people use the observability of actors’ behavior as a proxy to infer actors’ motivation. When people know actors’ motivation (as in the design of Experiments investigated in this analysis), however, this information determines their trait judgment, regardless of the observability of actors’ behavior.

## GENERAL DISCUSSION

Across three analyses martialing data from 14 experiments (seven preregistered, total *N* = 9,360), we provide robust evidence of *virtue discounting*. In brief, we show that when actors’ behavior is observable, people are less likely to attribute this behavior to actors’ virtue. In Analysis 1—which includes a meta-analysis of all experiments we ran—we show that observability causes virtue discounting (supporting our first preregistered hypothesis), and that this effect is larger in the context of generosity compared to fairness (as impartiality; supporting our second preregistered hypothesis). In Analysis 2, we provide suggestive evidence that participants’ motivational inferences mediate a large portion (72.6%) of the effect of observability on their ratings of actors’ trait virtue (supporting our third preregistered hypothesis). In Analysis 3, we show that when we experimentally manipulate actors’ motivation, observability loses its significant effect on participants’ judgments of actors’ trait virtue (providing stronger evidence supporting our third preregistered hypothesis). We now consider the contributions of our findings to the empirical literature as well as limitations of the present investigation. Finally, we conclude with practical implications for effective prosocial advocacy.

### Contributions to the Literature

Consistent with the majority of studies demonstrating virtue discounting (e.g., Newman & Cain, 2014), we show robust evidence of this effect in the context of *generosity* (Analysis 1). To this literature, the present investigation adds novel evidence that virtue discounting also occurs in the context of *fairness* (as impartiality), though to a lesser extent. Our investigation of motivational inferences as a mechanism of observability on ratings of trait virtue (Analysis 2) yield insights into: 1) the structure of motivational inferences; 2) a relatively novel motivational construct; and 3) mechanistic explanations of virtue discounting via motivational inferences.

First, following prior work (e.g., Lin-Healy & Small, 2012), we hypothesized that virtue discounting will be explained by observers attributing selfish motivations to publicly virtuous actors. Our exploratory factor analysis of motivational inference items yielded two factors that we labeled *principled* and *reputation-signaling*, and although these factors intuitively seem like conceptual opposites, they are only moderately negatively correlated (*r* = −.46). It may be compelling to broadly conceptualize the construct of “motivation” as a bipolar scale with the endpoints “selfish” and “selfless”, but here, we show that the conceptually “selfish” *reputation-signaling* motivation factor and the conceptually “selfless” *principled* motivation factor are less strongly (though still negatively) correlated than one might expect. It is intuitively plausible that actors might be motivated both to help others *and* to have others think well of them. Future work might therefore eschew a simplistic unidimensional conceptualization of motivational inferences, instead taking a more pluralistic approach and extending the present work to include other motivations for prosocial behavior suggested by the literature (Narvaez & Snow, 2019; Reiss & Havercamp, 1998).

Second, we were surprised to find that our *norm-signaling* motivational inference item was not uniquely captured by either of the two factors resulting from our exploratory factor analysis. One previous study has demonstrated the importance of norm-signaling motivational inferences in explaining perceptions of individuals sharing their gender pronouns (Kodipady et al., 2021), although we are unaware of other work exploring this construct. Although it might be argued that the mediation demonstrated by this item (i.e., explaining the effect of observability on trait ratings) was relatively minor (−7.3%), it is worth noting that this was roughly equivalent in magnitude to the effect of our *reputation-signaling* motivation factor (9.3%; technically, an “opposing mediation”; Kenny et al., 1998), which represents the most frequently cited motivational explanation of virtue signaling in previous work.

Third, perhaps the most puzzlingly counterintuitive finding we present is that virtue discounting is largely explained by observers’ inferences that public actors have lower *principled* motivations (accounting for 72.3% of the effect), and that *reputation-signaling* inferences mediate only 9.3% of this effect. Building on prior work, we expected that the effect of observability on virtue discounting would be explained by inferred selfish motivations; instead, it appears that it is actually a decrease in (conceptually “selfless”) *principled* motivational inferences, rather than an increase in (conceptually “selfish”) *reputation-signaling* motivational inferences that explains virtue discounting in our paradigm. We rule out a possible alternative explanation, that *reputation-signaling* motivational inferences mediate the effect of *principled* motivational inferences on trait ratings (see Supplement Section 7). Still, we believe more research is needed before placing great confidence in this conclusion. For example, previous demonstrations of virtue discounting often employ within-subjects comparisons of public vs. private actors (Lin-Healy & Small, 2012), but we compare public vs. private actors in between-subjects designs. Consistent with the literature on “joint vs. separate evaluation” (Hsee et al., 1999), it could be the case that *reputation-signaling* motivational inferences would emerge as the primary mechanism in a within-subjects design (see, e.g., a paradigm eliciting such reversals of moral judgment; McManus et al., 2020).

Although more research is needed to better understand which specific motivations contribute to virtue discounting, our results from Analysis 3 strengthen our interpretation that motivational inferences can explain the effect of observability in virtue discounting. Additional insight about the role of motivational inferences in virtue discounting is provided by comparing our results from Analysis 3 with our “baseline” (i.e., no observability information) conditions in Analysis 1. Taken together, these results suggest that people assume that actors have *principled* motivations, both when these actors’ behavior is conducted privately and also when information about the observability of their behavior is absent. It is unclear whether this pattern of results might be attributed to a simple “behavior-motivation congruency heuristic” (i.e., perhaps people tend to think that the valence of actors’ motivation matches their behavior) or reflects some sort of generalized trust (i.e., people assume others have good intentions).

Across our analyses, it is interesting to note that the relatively limited information we provide about actors’ behavior is sufficient to cause participants to rate actors as generally virtuous. Although observability reduces participants’ ratings of actors’ trait virtue—and explicitly stipulating actors’ reputation-signaling motivation roughly doubles that effect—participants still qualitatively rate actors positively. These results provide some evidence of people’s general willingness to charitably interpret others’ prosocial behavior. In light of this, it may provide justification—via promoting accurate assessment of others’ moral character and counteracting exploitation by manipulative actors—for some people to express skepticism in reaction to others’ observable prosocial behavior, including posting their good deeds on social media (i.e., speculating about others’ “virtue signaling”).

We conclude these considerations by contrasting our results with a puzzlingly divergent set of results. In many situations, observers react to virtuous behavior with positive valence emotions (see work on, e.g., elevation; Haidt, 2003), and further, that people often emulate virtuous behavior when they observe it (Thomson & Siegel, 2017). Yet, beyond the virtue discounting literature we discuss here, other work also suggests that such “do-gooders” are frequently derogated for their morally-motivated behavior (e.g., Sparkman & Attari, 2020). We propose a fascinating question for future research to explore: what individual- and/or situational-factors moderate such celebration (Bai et al., 2019) versus derogation (Minson & Monin, 2012) responses in social comparison (Mussweiler, 2003)?

### Limitations

The present investigation is not without shortcomings. We believe some caution is warranted regarding the mechanistic account of the effect of observability on trait virtue through motivational inferences given a comparison of our results across Analyses 2 and 3. In Analysis 3, when we stipulate that virtuous actors have *reputation-signaling* (and not *principled*) motivations, participants’ ratings of actors’ trait virtue are reduced to a greater extent than when we merely describe public actors (with no motive stipulated). Our manipulation of actor motivation thus may have been an overdetermination of our proposed mechanism, which could imply, for example, that potential mechanisms we failed to consider have a milder effect on trait ratings, or that there are individual differences in our proposed motivational inference mechanism. It could also be the case that observers infer that public actors have mixed motives, e.g., *both* principled and reputation-signaling motivations (see Analysis 2, correlation of these factors, *r* = −.46), which is obscured by our manipulations in Analysis 3 (e.g., we describe actors having reputation-signaling *but not* principled motivations).

Although we provide evidence that observers’ motivational inferences are a mechanism of virtue discounting, we do not claim that this is the *only* mechanism of virtue discounting. For example, the “bottom-up” manner in which the behaviors we used as stimuli were generated (Kraft-Todd et al., 2022) involved participants rating a set of behaviors expressing each virtue on 9 dimensions (e.g., descriptive normativity). It could be the case that another mechanism of virtue discounting are these underlying dimensions; i.e., if prototypical behaviors expressing virtues are systematically perceived differently along these (as well as other) dimensions, such variation could explain differential virtue discounting. Consider, for example, one dimension used in this prior work: the potential for actors to engage in these behaviors anonymously. If behaviors that prototypically express some virtues are lower on this dimension (i.e., they are necessarily publicly observable) than those that prototypically express other virtues, we might expect less discounting of the former compared to the latter. This is because actors’ discretion about observability in the latter case provides observers an opportunity to infer the motivations for actors’ choice (i.e., to do these behaviors publicly), which might include reputation-signaling. Future work might therefore explore the contribution of such underlying dimensions of behaviors prototypically expressing virtues as another potential mechanism of virtue discounting.

Additionally, we provide two empirical hypotheses among our motivations for our *differential virtue discounting* hypothesis that we do not test here. First, that perceived reputation management (through publicly observable virtuous behavior) will negatively impact observers’ perceptions of actors’ virtue more strongly in cases of “morally motivated deviance” (Cramwinckel et al., 2015) compared to conformity with social norms (Ohtsuki & Iwasa, 2006). Although we believe that the results of our “baseline condition” experiments (see Analysis 1) are consistent with this account—with these reasons for virtue discounting instantiated, respectively, in our generosity and fairness (as impartiality) conditions—directly testing this hypothesis represents a promising direction for future work. Second, we posited that virtue discounting is less likely to affect categorical norms (as with fairness/impartiality) than continuous norms (as with generosity). Although we believe this is beyond the scope of the current work, it represents another specific empirical hypothesis that we encourage future work to explore.

The generalizability of our findings may be limited because we investigated only two virtues, measured only six items of motivational inference, and our methods relied on exclusively hypothetical vignette scenarios administered to convenience samples. There is a substantial literature investigating other virtues (e.g., Peterson & Seligman, 2004)—and despite ambiguity in virtue concepts and their operationalization (McGrath, 2014)—we leave it to future work to examine whether the virtue discounting effect generalizes to other virtues (see, e.g., research on trustworthiness; Jordan, Hoffman, Bloom, et al., 2016). Similarly, we also recognize that motivation (like virtue) is a multidimensional construct (e.g., Reiss & Havercamp, 1998) and that previous work has suggested that distinct virtues are driven by distinct motivations (Narvaez & Snow, 2019). Future work might also measure a greater range of motivations for each virtue (see, e.g., Kodipady et al., 2021). For example, motivational inferences relevant to the present work that future research might explore are the degree to which observers believe that actors were motivated to: 1) demonstrate their moral superiority; 2) impose their values on others; and 3) manipulate others into behaving similarly (the latter two representing a negative interpretation of our “norm-signaling” item). Due to the limitations of drawing generalizable conclusions about moral judgment from hypothetical vignette studies (Feldman-Hall et al., 2012) and online convenience samples (Simons et al., 2017), we encourage future work to employ more diverse methods.

We also expect that different cultures may discount virtue differently. Fundamental to our definition of virtue is that the value of a trait that constitutes virtue derives from the social norms of the culture under investigation. Therefore, in addition to merely replicating our results in non-WEIRD samples (Henrich et al., 2010), we propose that our results are likely to vary in accordance with the value that different cultures place on different virtues. Beyond considering “culture” at such a broad level of analysis (e.g., nationality), we further encourage future work to consider narrower social identities (e.g., workplace) that might inform group members’ perception of virtues. Because individuals’ perception of virtues may be informed by social norms belonging to any of the social groups with which they identify—and comprehensively documenting these may be beyond the scope of any particular investigation—we finally suggest that future studies exploring perceptions of virtue incorporate measures of individual differences in participants’ motivational inferences. One source of variation might be people’s valuation of the virtues they are asked to make judgments about in others; e.g., in the present context, such data might show that individuals who more highly value particular virtues (e.g., generosity) are more likely to discount publicly observable behavior expressing those virtues. Another source of variation might be broader measures of sociability; e.g., people who are generally less trusting of others may be generally more skeptical of the motivations for others’ prosocial behavior.

In addition to these specific limitations, we also believe that our studies provide directions for future research that are beyond the scope of the current work. For example, we exclusively explored *virtue discounting* in the context of virtuous (i.e., morally good) behavior, although future work might consider exploring this phenomenon in the context of *unvirtuous* (i.e., morally bad) behavior.

### Practical Implications and Conclusion

Finally, our findings yield insights about effective advocacy of real-world virtuous behaviors. We started with a paradox of public prosociality: while others can only infer your virtue and learn from your prosocial example by observing your behavior, they simultaneously may be less likely to attribute your behavior to your virtue when your behavior is observable. Our results are consistent with this paradox of public prosociality, particularly when we consider the “opposing mediation” (Kenny et al., 1998; i.e., the negative mediation effect) of our *norm-signaling* item on trait ratings. To reiterate: the effect of observability on motivation inference is in the same direction for *norm-signaling* and *reputation-signaling* motivations, whereas the effect of motivation inference on trait virtue is in the same direction for *norm-signaling* and *principled* motivations. In other words, observable actors can be perceived positively if observers infer that actors have *norm-signaling* motivations; yet, observably virtuous actors also can be perceived negatively if observers infer that actors have low *principled* motivations (and also high *reputation-signaling* motivations). This pattern of results suggests that, to be perceived positively and potentially influence observer behavior, actors should consider how to convincingly convey *norm-signaling* motivations *without* engendering perceptions of low *principled* motivations (and also high *reputation-signaling* motivations).

Developing effective strategies for such communication is crucial for real-world advocates, and one hint of how this might be accomplished is provided by recent work on sharing gender pronouns in the workplace (Kodipady et al., 2021). One finding from this work is that a transgender (compared to a cisgender) advocate is more likely to be perceived as norm-signaling and less likely to be perceived as reputation-signaling. These results suggest that recruiting advocates whose identity is congruent with the target issue may mitigate reputation-signaling attributions. This work leaves open the question of how non-identity-congruent (in the context of that work, cisgender) advocates can be effective communicators, highlighting another direction for future research related to “effective ally-ship” (Radke et al., 2020).

Further, following our preceding discussion regarding a costly signaling account of virtue discounting, it could be the case that for virtues where “selfish” and “selfless” motivations are perceived more dichotomously, actors need to more clearly demonstrate their “selfless” motivations to avoid observers discounting their virtue. This concern echoes our second preregistered hypothesis (“differential virtue discounting”), which suggests that practitioners (as well as researchers) should carefully examine which virtues they are displaying in their advocacy, and consider whether these are particularly susceptible to virtue discounting and diluting their overall message.

If prosocial advocates can credibly demonstrate that they are motivated by *principles* and *norm-signaling*, and not by *reputation-signaling*—particularly in the context of some virtues (e.g., generosity)—they may be able to resolve the paradox of public prosociality. One might wonder, “how can prosocial advocates credibly demonstrate their motivation?” In addition to a direction for future research, we leave this question to prosocial advocates as a prompt for reflection. The answer will undoubtedly be contingent on the individual, audience, and setting, but finding it may be a key to catalyzing the contagion of prosociality.

## ACKNOWLEDGMENTS

We would like to thank the members of the Morality Lab and the reviewers for their generous feedback.

## FUNDING INFORMATION

This research was made possible by funding by the John Templeton Foundation, The Virtue Project at Boston College, and NSF award #1627157.

## OPEN PRACTICES

Preregistrations were conducted for: E8 (https://aspredicted.org/FLG_VRD), E9 (https://aspredicted.org/NUP_ESB), E10 (https://aspredicted.org/FCO_HXM), E11 (https://aspredicted.org/2Y2_YKT), E12 (https://aspredicted.org/AES_HGO), E13 (https://aspredicted.org/G6S_S35), and E14 (https://aspredicted.org/SHW_9PN). All data and code are publicly available at: https://osf.io/sud3m/?view_only=380a169770b9474f93d2b5b73adc7410.

## SUPPORTING INFORMATION

Details on our supplementary materials are as follows:

- Supplementary Materials: We provide supplementary materials—including supplementary analyses and complete experimental instructions—in a Word file available for download from the journal’s website as a separate file named “Virtue Discounting Supplemental Materials.docx”.
- Code and Data Repository: The code and data used for our statistical analyses are available at: https://osf.io/sud3m/?view_only=380a169770b9474f93d2b5b73adc7410.

## Notes

^1^ We note that although we treat *moral rule* and *virtue identification* motivations as “socially-independent”, this may only apply to the actors’ proximate psychology, as both motivations are likely shaped through cultural norm internalization (Henrich, 2016).^2^ Note that we only tested generosity in E10 so this experiment is not included in this analysis.
