# Supplementary material for: Virtue Discounting: Observability Reduces Moral Actors’ Perceived Virtue
Source: Open Mind (Camb). 2023 Jul 26;7:460–82. doi: 10.1162/opmi_a_00085 (PMC10449397; doi:10.1162/opmi_a_00085)
Supplement: Supplementary file 1 [file opmi-07-460-s001.docx]

**Supplemental Materials**

***for***

Virtue Discounting:

Observability Reduces Moral Actors’ Perceived Virtue

Contents

[Section 1. Summary of experiment characteristics and participant demographics 2](#_Toc122518768)

[Supplementary Table 1. Experiment characteristics. 2](#_Toc122518769)

[*Shown are preregistrations, sample sizes, dependent measures, and conditions in all experiments.* 2](#_Toc122518770)

[Supplementary Table 2. Basic demographics across all experiments. 2](#_Toc122518771)

[Section 2. Justifications for changes in design across experiments 3](#_Toc122518772)

[Section 3. Robustness analyses. 4](#_Toc122518773)

[Supplementary Table 3. Bivariate correlations between primary dependent measures (moral goodness ratings and trait ratings) by experiment. 4](#_Toc122518774)

[Supplementary Table 4. Virtue discounting results by experiment (moral goodness ratings). 4](#_Toc122518775)

[Supplementary Table 5. Virtue discounting results by experiment (trait ratings). 4](#_Toc122518776)

[Supplementary Table 6. Differential virtue discounting results by experiment (moral goodness ratings). 4](#_Toc122518777)

[Supplementary Table 7. Differential virtue discounting results by experiment (trait ratings). 4](#_Toc122518778)

[Supplementary Table 8. Discounting results by experiments with exclusions 5](#_Toc122518779)

[Supplementary Table 9. Virtue discounting results by subset (trait ratings). 5](#_Toc122518780)

[Supplementary Table 10. Differential virtue discounting results by subset (trait ratings). 5](#_Toc122518781)

[Analysis 3: Results omitting virtue manipulation. 6](#_Toc122518782)

[Section 4. Comparison of observability manipulations with “baseline” no observability information condition 7](#_Toc122518783)

[Supplementary Figure 1. Participants perceive (generous) actors described without observability information similar to privately virtuous actors. 7](#_Toc122518784)

[Section 5: Mediated moderation analysis 8](#_Toc122518785)

[Supplementary Figure 2. Differential virtue discounting is explained by observers’ inferences that actors have lower authentic motivations (for both generosity and impartiality). 8](#_Toc122518786)

[Section 6: Correlation among motivational inference items 10](#_Toc122518787)

[Supplementary Table 11. Pairwise correlations (*r*) between all six motivational inference items measured. 10](#_Toc122518788)

[Section 7: Alternative mediation models 11](#_Toc122518789)

[Supplementary Figure 3. Alternative specifications of mediation models explaining the effect of observability on trait ratings via motivational inferences. 11](#_Toc122518790)

[Supplementary Table 12. Regression results implied by alternative mediation model specifications of the effect of observability on trait ratings via motivational inferences. 12](#_Toc122518791)

[Section 8. Complete experimental instructions 13](#_Toc122518792)

[*Common elements across all experiments* 13](#_Toc122518793)

[*Manipulations* 17](#_Toc122518794)

[*Dependent measures* 27](#_Toc122518795)

# **Section 1. Summary of experiment characteristics and participant demographics**

## **Supplementary Table 1. Experiment characteristics.**

## *Shown are preregistrations, sample sizes, dependent measures, and conditions in all experiments.*

## **Supplementary Table 2.** Basic demographics across all experiments.

# **Section 2. Justifications for changes in design across experiments**

Throughout our investigation, we iterated on our experimental design. We document the progression of these changes to each design element below. After collecting data for E7, we felt sufficiently confident in our design to begin preregistering subsequent designs and analyses. We include all data in the analyses presented in the main text to alleviate file-drawer concerns as well as to demonstrate the robustness of our results.

Beginning with E1 (and continuing through E6), we wanted to provide the strongest possible test of our observability manipulation. In these experiments, we therefore used a “kitchen sink” approach, simultaneously manipulating observability (public vs. private) as well as actors’ motivation, matched to the motivational inference we believe people typically infer for each condition (selfish vs. selfless, respectively). Because we were interested in measuring participants’ inferences of actors’ motivations in subsequent experiments, we did not stipulate actor motivation thereafter (except E10 and E14, in which we crossed our observability manipulation with a manipulation of actors’ motivations). Prior to E10, we found that participants engaged in virtue discounting for generous but not impartial actors, and so we only included generosity conditions in E10.

The sets of (three) behaviors we employed as stimuli also varied across experiments. In E1-3, we used no example behaviors (instead, participants only read virtue definitions). In E4 and E5, we used experiment-generated behaviors. In E6-14, we used participant-generated behaviors experiments (following the procedure described in our previous work; Kraft-Todd et al., 2022)^[[1]](#footnote-1)^. The set of behaviors used in E6-10 were approximately representative of differences in perceptions between generosity and impartiality as rated by an independent sample of participants across 9 underlying features (Kraft-Todd et al., 2022). The set of behaviors used in E11 and E12 were selected because they were matched across ratings of these features (i.e. they were not different between virtues). The set of behaviors used in E13 and E14 were selected because they were precisely representative of the virtues across ratings of these features (as opposed to those we used in E6-10 which were only approximately representative).

# **Section 3. Robustness analyses.**

For all tables:

- Hypothesis test effects bolded
- Standard errors in parentheses
- *** p<0.01, ** p<0.05, * p<0.1

## **Supplementary Table 3.** Bivariate correlations between primary dependent measures (moral goodness ratings and trait ratings) by experiment.

| *Experiment* | 1 | 2 | 3 | 4 | 5 | 6 | 7 | 8 | 9 | 10 | 11 | 12 | 13 | 14 |
| --- | --- | --- | --- | --- | --- | --- | --- | --- | --- | --- | --- | --- | --- | --- |
| *r* | .68*** | .67*** | .66*** | .75*** | .78*** | .79*** | .58*** | .53*** | .58*** | .87*** | .57*** | .75*** | .69*** | .80*** |

## **Supplementary Table 4.** Virtue discounting results by experiment (moral goodness ratings).

|  | E1 | E2 | E3 | E4 | E5 | E6 | E7 | E8 | E9 | E10 | E11 | E12 | E13 | E14 |
| --- | --- | --- | --- | --- | --- | --- | --- | --- | --- | --- | --- | --- | --- | --- |
| Observability | **-19.90***** | **-18.41***** | **-15.97***** | **-18.44***** | **-19.93***** | **-24.80***** | **-5.356***** | **-5.790***** | **-5.814***** | **-4.635***** | **-1.820** | **-1.437** | **-8.812***** | **-7.418***** |
| (1=public) | (1.724) | (1.703) | (1.776) | (1.725) | (1.648) | (1.697) | (1.708) | (1.596) | (1.671) | (1.794) | (1.635) | (1.438) | (0.817) | (0.985) |
| Constant | 83.04*** | 82.94*** | 80*** | 83.15*** | 83.17*** | 83.95*** | 78.45*** | 81.07*** | 79.68*** | 76.42*** | 80.51*** | 77.34*** | 82.48*** | 75.75*** |
|  | (1.217) | (1.204) | (1.256) | (1.220) | (1.173) | (1.198) | (1.208) | (1.127) | (1.194) | (1.273) | (1.150) | (1.021) | (0.581) | (0.695) |
| Observations | 389 | 394 | 394 | 388 | 393 | 393 | 388 | 383 | 386 | 663 | 394 | 377 | 1,770 | 2,260 |
| R-squared | 0.256 | 0.230 | 0.171 | 0.228 | 0.272 | 0.353 | 0.025 | 0.033 | 0.031 | 0.010 | 0.003 | 0.003 | 0.062 | 0.024 |
| Prereg? | N | N | N | N | N | N | N | Y | Y | Y | Y | Y | Y | Y |

## **Supplementary Table 5.** Virtue discounting results by experiment (trait ratings).

|  | E1 | E2 | E3 | E4 | E5 | E6 | E7 | E8 | E9 | E10 | E11 | E12 | E13 | E14 |
| --- | --- | --- | --- | --- | --- | --- | --- | --- | --- | --- | --- | --- | --- | --- |
| Observability | **-21.66***** | **-20.37***** | **-19.72***** | **-21.73***** | **-25.44***** | **-28.72***** | **-3.876**** | **-6.017***** | **-5.817***** | **-3.374*** | **-1.387** | **-0.910** | **-7.363***** | **-7.057***** |
| (1=public) | (1.979) | (2.175) | (1.868) | (1.878) | (1.817) | (1.818) | (1.731) | (1.788) | (1.694) | (1.980) | (1.859) | (1.548) | (0.789) | (1.054) |
| Constant | 88.34*** | 87.43*** | 87.99*** | 87.35*** | 86.76*** | 88.71*** | 83.80*** | 83.80*** | 84.98*** | 74.97*** | 78.38*** | 76.54*** | 85.14*** | 77.26*** |
|  | (1.398) | (1.538) | (1.321) | (1.328) | (1.293) | (1.284) | (1.224) | (1.263) | (1.210) | (1.405) | (1.308) | (1.099) | (0.561) | (0.743) |
| Observations | 389 | 394 | 394 | 388 | 393 | 393 | 388 | 383 | 386 | 663 | 394 | 377 | 1,767 | 2,260 |
| R-squared | 0.236 | 0.183 | 0.221 | 0.258 | 0.334 | 0.389 | 0.013 | 0.029 | 0.030 | 0.004 | 0.001 | 0.001 | 0.047 | 0.019 |
| Prereg? | N | N | N | N | N | N | N | Y | Y | Y | Y | Y | Y | Y |

## **Supplementary Table 6.** Differential virtue discounting results by experiment (moral goodness ratings).

|  | E1 | E2 | E3 | E4 | E5 | E6 | E7 | E8 | E9 | E10 | E11 | E12 | E13 | E14 |
| --- | --- | --- | --- | --- | --- | --- | --- | --- | --- | --- | --- | --- | --- | --- |
| Virtue | 7.128*** | 5.323** | 8.853*** | 8.451*** | 6.399*** | 1.850 | 6.806*** | 4.687** | 7.080*** |  | 3.774 | 2.136 | 11.00*** | 3.782*** |
| (1=generous) | (2.404) | (2.343) | (2.447) | (2.398) | (2.298) | (2.387) | (2.397) | (2.247) | (2.366) |  | (2.294) | (2.040) | (1.127) | (1.388) |
| Observability | -14.15*** | -10.63*** | -7.747*** | -12.04*** | -13.05*** | -21.27*** | -1.453 | -3.490 | -2.710 | -4.635*** | -1.245 | -1.660 | -5.507*** | -5.303*** |
| (1=public) | (2.410) | (2.343) | (2.441) | (2.392) | (2.287) | (2.399) | (2.415) | (2.247) | (2.355) | (1.794) | (2.312) | (2.035) | (1.119) | (1.394) |
| Virtue*Obs | **-11.46***** | **-15.64***** | **-16.54***** | **-12.97***** | **-13.73***** | **-7.033**** | **-7.717**** | **-4.601** | **-6.016*** |  | **-1.210** | **0.407** | **-6.526***** | **-4.290**** |
|  | (3.403) | (3.313) | (3.460) | (3.391) | (3.230) | (3.380) | (3.390) | (3.181) | (3.312) |  | (3.261) | (2.874) | (1.585) | (1.968) |
| Constant | 79.45*** | 80.24*** | 75.60*** | 79.06*** | 79.94*** | 83.01*** | 75.01*** | 78.73*** | 76.04*** | 76.42*** | 78.63*** | 76.27*** | 76.96*** | 73.89*** |
|  | (1.704) | (1.669) | (1.726) | (1.669) | (1.633) | (1.701) | (1.703) | (1.589) | (1.695) | (1.273) | (1.618) | (1.439) | (0.798) | (0.972) |
| Observations | 389 | 394 | 394 | 388 | 393 | 393 | 388 | 383 | 386 | 663 | 394 | 377 | 1,770 | 2,260 |
| R-squared | 0.279 | 0.275 | 0.217 | 0.259 | 0.305 | 0.362 | 0.045 | 0.044 | 0.053 | 0.010 | 0.013 | 0.010 | 0.117 | 0.028 |

*NOTE: E10 only included generosity (not impartiality).*

## **Supplementary Table 7.** Differential virtue discounting results by experiment (trait ratings).

|  | E1 | E2 | E3 | E4 | E5 | E6 | E7 | E8 | E9 | E10 | E11 | E12 | E13 | E14 |
| --- | --- | --- | --- | --- | --- | --- | --- | --- | --- | --- | --- | --- | --- | --- |
| Virtue | 2.879 | 2.333 | 5.726** | 2.890 | 1.747 | 0.141 | 7.871*** | 8.333*** | 5.624** |  | 3.783 | 4.747** | 10.51*** | 4.070*** |
| (1=generous) | (2.793) | (3.047) | (2.616) | (2.639) | (2.584) | (2.542) | (2.419) | (2.496) | (2.409) |  | (2.615) | (2.179) | (1.094) | (1.484) |
| Observability | -18.46*** | -14.72*** | -13.96*** | -17.03*** | -22.65*** | -24.58*** | 1.103 | -1.948 | -2.017 | -3.374* | 0.552 | -0.652 | -2.810*** | -4.383*** |
| (1=public) | (2.800) | (3.047) | (2.609) | (2.632) | (2.571) | (2.555) | (2.438) | (2.496) | (2.397) | (1.980) | (2.636) | (2.174) | (1.087) | (1.490) |
| Virtue*Obs | **-6.384** | **-11.40***** | **-11.57***** | **-9.352**** | **-5.563** | **-8.273**** | **-9.838***** | **-8.137**** | **-7.460**** |  | **-3.896** | **-0.585** | **-9.033***** | **-5.393**** |
|  | (3.955) | (4.309) | (3.700) | (3.732) | (3.631) | (3.600) | (3.421) | (3.534) | (3.372) |  | (3.718) | (3.070) | (1.538) | (2.105) |
| Constant | 86.90*** | 86.25*** | 85.14*** | 85.95*** | 85.87*** | 88.64*** | 79.82*** | 79.64*** | 82.10*** | 74.97*** | 76.50*** | 74.18*** | 79.85*** | 75.26*** |
|  | (1.980) | (2.171) | (1.845) | (1.837) | (1.836) | (1.811) | (1.719) | (1.765) | (1.726) | (1.405) | (1.845) | (1.537) | (0.776) | (1.039) |
| Observations | 389 | 394 | 394 | 388 | 393 | 393 | 388 | 383 | 386 | 663 | 394 | 377 | 1,767 | 2,260 |
| R-squared | 0.242 | 0.202 | 0.240 | 0.271 | 0.338 | 0.405 | 0.041 | 0.057 | 0.045 | 0.004 | 0.007 | 0.023 | 0.095 | 0.023 |

*NOTE: E10 only included generosity (not impartiality).*

## **Supplementary Table 8.** Discounting results by experiments with exclusions

|  | E10 | | E11 | | | | E13 | | | | E14 | | | |
| --- | --- | --- | --- | --- | --- | --- | --- | --- | --- | --- | --- | --- | --- | --- |
| DV: | Good | Trait | Good | Trait | Good (int) | Trait  (int) | Good | Trait | Good (int) | Trait  (int) | Good | Trait | Good (int) | Trait  (int) |
| Virtue (1=generous) |  |  |  |  | 4.118*  (2.367) | 3.858  (2.705) |  |  | 11.03***  (1.190) | 10.04***  (1.160) |  |  | 4.289***  (1.525) | 3.711**  (1.650) |
| Observability | -4.864** | -3.877* | -1.540 | -1.169 | -1.049 | 0.677 | -8.699*** | -7.263*** | -5.500*** | -2.817** | -8.068*** | -8.138*** | -4.930*** | -4.454*** |
| (1=public) | (1.975) | (2.184) | (1.679) | (1.913) | (2.354) | (2.691) | (0.865) | (0.836) | (1.187) | (1.157) | (1.076) | (1.164) | (1.532) | (1.657) |
| Virtue*Obs |  |  |  |  | -1.030 | -3.752 |  |  | -6.371*** | -8.852*** |  |  | -6.218*** | -7.250*** |
|  |  |  |  |  | (3.347) | (3.826) |  |  | (1.679) | (1.635) |  |  | (2.149) | (2.325) |
| Constant | 75.90*** | 74.65*** | 80.36*** | 78.17*** | 78.33*** | 76.27*** | 82.55*** | 85.22*** | 77.03*** | 80.18*** | 75.90*** | 77.71*** | 73.76*** | 75.86*** |
|  | (1.403) | (1.551) | (1.187) | (1.353) | (1.660) | (1.898) | (0.614) | (0.593) | (0.842) | (0.822) | (0.764) | (0.827) | (1.077) | (1.165) |
| Exclusions? | Y | Y | Y | Y | Y | Y | Y | Y | Y | Y | Y | Y | Y | Y |
| Observations | 559 | 559 | 378 | 378 | 378 | 378 | 1,579 | 1,576 | 1,579 | 1,576 | 1,851 | 1,851 | 1,851 | 1,851 |
| R-squared | 0.011 | 0.006 | 0.002 | 0.001 | 0.015 | 0.006 | 0.060 | 0.046 | 0.117 | 0.090 | 0.029 | 0.026 | 0.034 | 0.031 |

*NOTE: E10 only included generosity (not impartiality). All exclusions were preregistered.*

For Tables 9 and 10:

- Model 1: No example behaviors used as stimuli (E1-3)
- Model 2: Experimenter-generated example behaviors used as stimuli (E4 and E5)
- Model 3: Participant-generated example behaviors used as stimuli (E6-14)
- Model 4: Conditions in which we stipulated actor motivation (E1-6 and *principled* and *reputation-signaling* conditions in E10 and E14)
- Model 5: Conditions in which we did not stipulate actor motivation (E7-9, E11-13, and “no motivation stipulated” conditions in E10 and E14)
- Model 6: Preregistered experiments (E8-14)
- Model 7: Non-preregistered experiments (E1-7)

## **Supplementary Table 9.** Virtue discounting results by subset (trait ratings).

|  | Model 1 | Model 2 | Model 3 | Model 4 | Model 5 | Model 6 | Model 7 |
| --- | --- | --- | --- | --- | --- | --- | --- |
| Observability | **-20.50***** | **-23.61***** | **-6.987***** | **-15.41***** | **-5.550***** | **-5.811***** | **-20.21***** |
| (1=public) | (1.157) | (1.309) | (0.507) | (0.735) | (0.484) | (0.543) | (0.735) |
| Constant | 87.86*** | 87.05*** | 80.80*** | 80.70*** | 83.75*** | 80.11*** | 87.18*** |
|  | (0.818) | (0.929) | (0.359) | (0.518) | (0.344) | (0.384) | (0.520) |
| Observations | 1,183 | 781 | 7,016 | 4,308 | 4,672 | 6,235 | 2,745 |
| R-squared | 0.210 | 0.295 | 0.026 | 0.093 | 0.027 | 0.018 | 0.216 |

## **Supplementary Table 10.** Differential virtue discounting results by subset (trait ratings).

|  | Model 1 | Model 2 | Model 3 | Model 4 | Model 5 | Model 6 | Model 7 |
| --- | --- | --- | --- | --- | --- | --- | --- |
| Virtue | 3.639** | 2.305 | 4.685*** | 0.0120 | 8.061*** | 4.899*** | 3.358*** |
| (1=generous) | (1.624) | (1.849) | (0.719) | (1.039) | (0.679) | (0.770) | (1.033) |
| Observability | -15.54*** | -19.92*** | -3.892*** | -13.09*** | -1.885*** | -2.786*** | -15.75*** |
| (1=public) | (1.623) | (1.842) | (0.752) | (1.094) | (0.693) | (0.810) | (1.034) |
| Virtue*Obs | **-9.942***** | **-7.371***** | **-5.666***** | **-4.165***** | **-6.953***** | **-5.493***** | **-8.898***** |
|  | (2.296) | (2.607) | (1.016) | (1.475) | (0.955) | (1.089) | (1.460) |
| Constant | 86.03*** | 85.91*** | 78.25*** | 80.70*** | 79.50*** | 77.42*** | 85.49*** |
|  | (1.151) | (1.301) | (0.531) | (0.766) | (0.493) | (0.571) | (0.732) |
| Observations | 1,183 | 781 | 7,016 | 4,308 | 4,672 | 6,235 | 2,745 |
| R-squared | 0.223 | 0.303 | 0.032 | 0.096 | 0.056 | 0.024 | 0.227 |

## **Analysis 3: Results omitting virtue manipulation.**

We begin by conducting a regression analysis predicting trait ratings by the interaction of the observability manipulation (public vs. private), the actor motive manipulation (reputation-signaling vs. principled vs. none; coded as an indicator variable with “none” as the holdout condition), and our virtue manipulation (generosity vs. impartiality), with Experiment as a covariate. We observe that the three-way interaction terms are significant (virtue*observability*reputation-signaling: *p*=.004; virtue*observability*principled: *p*=.004). Because this result implies that the observability*motive interaction differs by virtue, we conduct two regression analyses (mirroring the previous specification) of this interaction disaggregated by virtue. These analyses reveal that, in both virtue conditions, the effect of observability on trait ratings is reduced in the *principled* actor motive conditions compared to the *no motive stipulated* conditions (*generosity*: *p*<.001; *impartiality*: *p*=.020). However, compared to the *no motive stipulated* conditions, the effect of observability on trait ratings is reduced in the *reputation-signaling* actor motive conditions for *generosity* (*p*<.001) but not for *impartiality* (*p*=.710).

When we omit the virtue manipulation from the analysis presented in the main text, we again find a significant interaction between observability and motive on trait ratings (*F*(2,8950)=26.51, *p*<.001, *d*=.15). When we do not stipulate actor motivation, we again observe *virtue discounting*; i.e. publicly virtuous actors are rated as less virtuous than privately virtuous actors (*contrast*=-5.60, 95% CI [-7.41, -3.78], *t*=-10.26, *p*<.001). Consistent with our hypothesis (preregistered in E10 and E14), we observe that publicly and privately virtuous actors are not rated differently on trait virtue when we stipulate that actors have *reputation-signaling* motivation (*contrast*=-2.54, 95% CI [-6.15, 1.06], *t*=-2.35, *p*=.357) or *principled* motivation (*contrast*=2.83, 95% CI [-.80, 6.45], *t*=2.60, *p*=.241).

# **Section 4.** **Comparison of observability manipulations with “baseline” no observability information condition**

Our aim in this analysis was to better understand the effect of our observability manipulation; specifically, how participants perceptions of our vignettes without observability information (i.e. whether actors did the behaviors in public or private) compare to our standard design (i.e. including observability information).

*Methods*

Using data from 2 online studies (E7 and E8; total *N*=1,159), we replicate Analysis 1, except that here, our design is a 2 (virtue: generosity vs. impartiality) x 3 (observability: public vs. private vs. no information) factorial design. We conduct a multivariate regression (including Experiment as a covariate) with the “no information” observability condition as the comparison group to test for how participants perceive this condition in comparison to the “public” and “private” observability conditions.

*Results*

We find a significant interaction between virtue and observability on trait ratings (*F*(2,1152)=7.66, *p*=.001). Compared to generous actors described without observability information, privately generous actors are not rated differently (*contrast*=-3.17, 95% CI [-8.65, 2.31], Scheffe’s *t*=-1.93, *p*=.571), while publicly generous actors are rated as significantly less virtuous (*contrast*=-6.23, 95% CI [-11.71, -.75], Scheffe’s *t*=-3.79, *p*=.014). In these experiments, impartial actors’ virtue was not discounted (comparing public to private observability conditions: *contrast*=-.43, 95% CI [-5.95, 5.09], Scheffe’s *t*=-.26, *p*=1.000), so this comparison cannot be made for impartiality. This result suggests that, absent observability information, participants perceive (generous) actors in a similar fashion to how they perceive privately (generous) actors, and that providing information that actors’ virtue was publicly observable leads to virtue discounting.


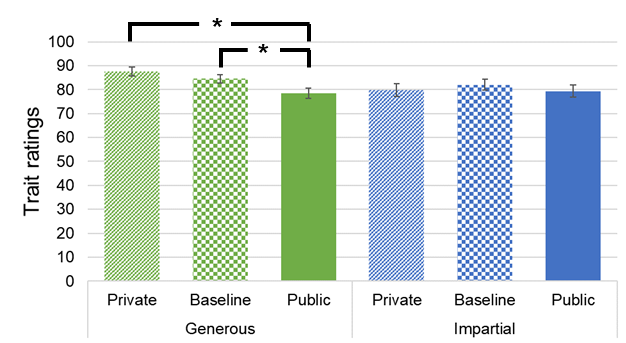


Supplementary Figure 1. Participants perceive (generous) actors described without observability information similar to privately virtuous actors.

Shown are means (with 95% CIs) of trait ratings (0-100 unmarked slider), as a function of whether the actor is said to engage in behaviors that are generous (green) or impartial (blue) and whether these behaviors are said to be displayed in public (solid), private (lines), or without observability information (checkered). Significant contrasts denoted with (*).

# **Section 5: Mediated moderation analysis**

Following from the results presented in Analysis 1, here we explore how motivational inferences mediate the virtue*observability interaction, i.e. accounting for the *differential virtue discounting* effect. We construct a structural equation model to test this (multiple) moderated mediation analysis, investigating the mediation of observability on traut ratings by the two motivational inference factor scores plus the norm-signaling item simultaneously for each virtue, controlling for Experiment and the covariance among mediators.

Participants infer that, compared to privately virtuous actors, publicly virtuous actors have significantly lower *principled* motivation (*generosity*: *b*=-.82, 95% CI [-.90, -.74], *p*<.001, see Figure 2a; *impartiality*: *b*=-.29, 95% CI [-.38, -.21], *p*<.001, see Figure 2b), and significantly higher *reputation-signaling* motivation (*generosity*: *b*=.85, 95% CI [.77, .94], *p*<.001; *impartiality*: *b*=.48, 95% CI [.40, .56], *p*<.001), and *norm-signaling* motivations (*generosity*: *b*=.28, 95% CI [.19, .37], *p*<.001; *impartiality*: *b*=.28, 95% CI [.19, .37], *p*<.001). Next, we find that participants’ trait ratings are significantly associated with their motivational inferences, such that higher *principled* (*generosity*: *b*=.53, 95% CI [.50, .56], *p*<.001; *impartiality*: *b*=.56, 95% CI [.53, .59], *p*<.001) and *norm-signaling* (*generosity*: *b*=.05, 95% CI [.02, .08], *p*<.001; *impartiality*: *b*=.05, 95% CI [.02, .08], *p*<.001) inferences are associated with higher trait ratings, while higher *reputation-signaling* inferences are associated with lower trait ratings (*generosity*: *b*=-.06, 95% CI [-.10, -.03], *p*<.001; *impartiality*: *b*=-.09, 95% CI [-.12, -.06], *p*<.001).


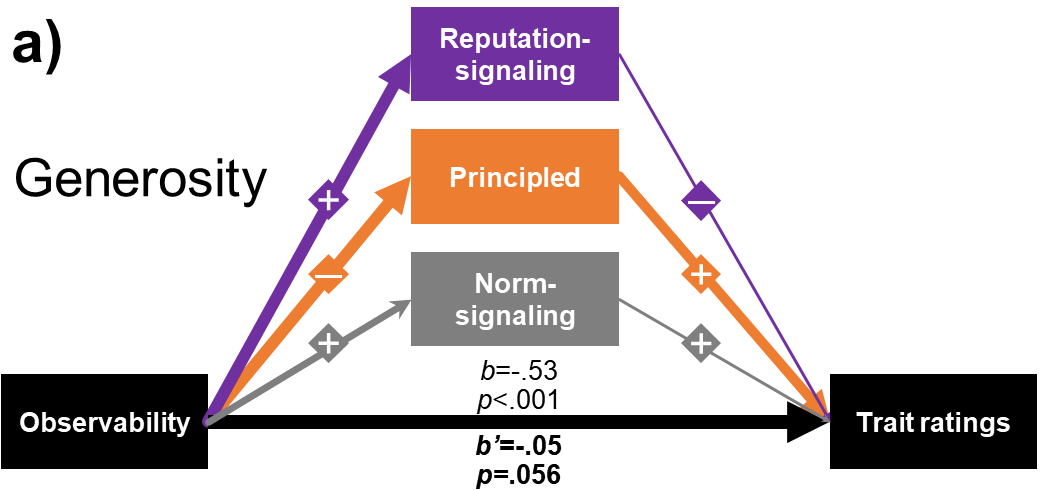

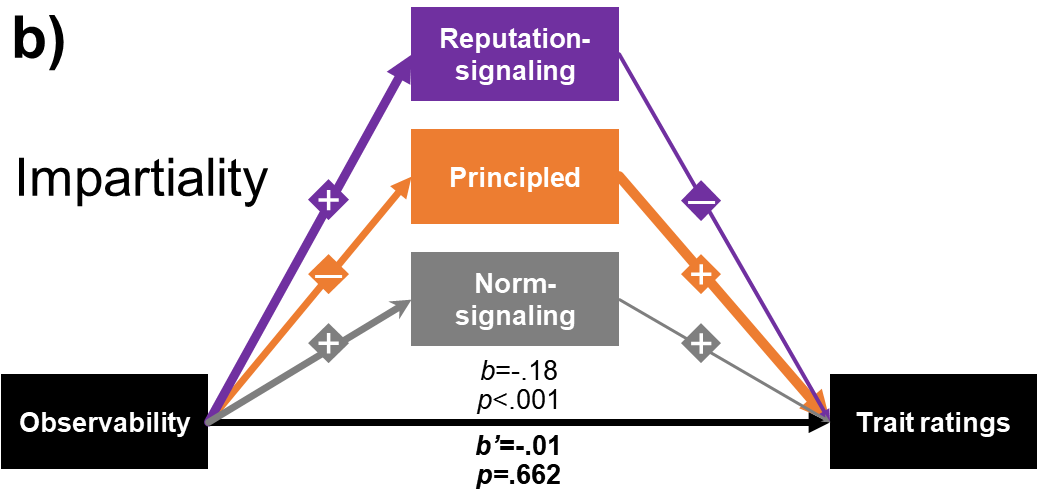


Supplementary Figure 2. Differential virtue discounting is explained by observers’ inferences that actors have lower authentic motivations (for both generosity and impartiality).

Shown are results from a generalized structural equation model (showing correlational multiple mediation) of the effect of observability on trait ratings by motivational inference factor scores and norm-signaling item visualized separately by virtue, i.e. **a)** generosity (*N*=2,050) and **b)** impartiality (*N*=2,037). Bottom arrow (black) represents the effect of the observability manipulation (public compared to private condition) on trait ratings with (b) and without (b’) mediators as covariates. From left-to-right, the first set of arrows represents the effect of the observability manipulation on mediators, and the second set of arrows represents the correlation of mediators with trait ratings. Line thickness represents correlation strength; “+” and “-” represent correlation direction; all variables standardized for this analysis.

Finally, we turn to the mediation results. Restating the *differential virtue discounting* result we present in Analysis 1, the total effect of observability on trait ratings is significant for both virtues such that participants rate public actors as less virtuous than private actors (*generosity*: *b*=-.53, 95% CI [-.60, -.46], *p*<.001; *impartiality*: *b*=-.18, 95% CI [-.25, -.11], *p*<.001). The direct effect of observability on trait ratings (i.e. accounting for indirect effects through motivational inferences) is both greatly reduced in magnitude and not significant for both virtues (*generosity*: *b’*=-.05, 95% CI [-.11, .001], *p*=.056; *impartiality*: *b’*=.01, 95% CI [-.04, .06], *p*=.662), implying full mediation (88.7% and 106.4% of the total effects, respectively). Calculating indirect effects as percent of total effect, *principled* motivation accounts for (*generosity*) 82.2% and (*impartiality*) 90.9% of this mediation, *reputation-signaling* motivation accounts for (*generosity*) 10.4% and (*impartiality*) 23.3%, and *norm-signaling* motivation accounts for (*generosity*) -2.7% and (*impartiality*) -7.8%.

Note that there are not qualitative differences in the motivational inferences driving virtue discounting of generosity compared to impartiality (i.e. it is not the case that different types of motivations explain virtue discounting for each virtue, only the degree to which observers infer these motivations). Consistent with *differential virtue discounting*, observability leads participants to make significantly stronger inferences of *principled* and *reputation-signaling* motivations for generous compared to impartial actors (i.e. the 95% CIs of these effects are non-overlapping); all other effects in this model are not significantly different.

# **Section 6: Correlation among motivational inference items**

## **Supplementary Table 11.** Pairwise correlations (*r*) between all six motivational inference items measured.

# **Section 7: Alternative mediation models**

The mediation model we present in Analysis 2 yields a potentially surprising result: 86.6% of the virtue discounting effect decrease in *principled* motivational inferences while only 12.7% of the effect was explained through an increase in *reputation-signaling* motivational inferences. Prior demonstrations of virtue discounting^[[2]](#footnote-2)^ typically cite “selfish” motivational inferences (conceptually similar to *reputation-signaling*) as the primary driver of this effect. Aligned with this explanation, it could be the case that the effect of *principled* motivational inferences are, in fact, mediated by *reputation-signaling* motivational inferences. To investigate whether this account described in previous work provides a more parsimonious explanation of our virtue discounting effect, we compare indices of model fit (AIC/BIC) for the model we specify in Analysis 2 (Supplementary Figure 3a) to alternatively specified mediation models (Supplementary Figure 3b).

First, we observe indices of model fit for the model we specify in Analysis 2 (a multiple mediation variant of PROCESS model 4; Hayes, 2013)^[[3]](#footnote-3)^: (AIC) 38,607; (BIC) 38,872. Next, we construct an alternative generalized structural equation model in which *principled* motivational inferences are mediated by *reputation-signaling* motivational inferences (a variant of PROCESS model 6) and observe indices of model fit: (AIC) 38,609; (BIC) 38,880. The model we specify in Analysis 2 is only slightly preferable to the alternative model based on a comparison of indices of model fit. We therefore additionally investigate the mediation results provided by the alternative model to aid our interpretation (Supplementary Table 12).

**
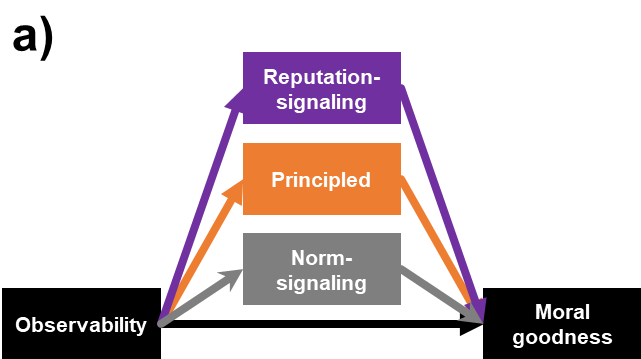

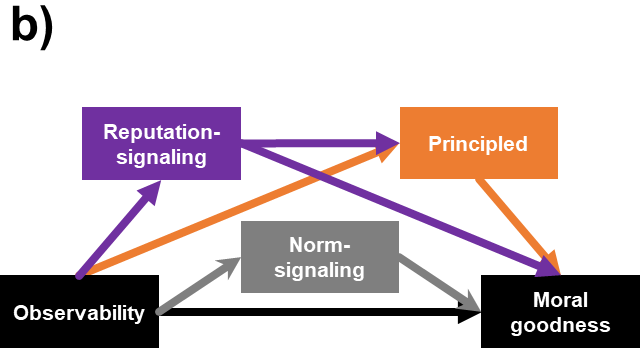
**

Supplementary Figure 3. Alternative specifications of mediation models explaining the effect of observability on trait ratings via motivational inferences.

Shown are conceptual diagrams of generalized structural equation models (showing correlational multiple mediation) of the effect of observability on trait ratings by motivational inference factor scores and norm-signaling item **a)** the model we present in Analysis 2 **b)** an alternative model (motivated by prior work) in which *principled* motivational inferences are mediated by *reputation-signaling* motivational inferences.

Using regression results implied by the alternative model (Supplementary Table 12; Columns 2, 3, and 5), we observe that *reputation-signaling* motivational inferences mediate 54.8% of the effect of *principled* motivational inferences on trait ratings. Despite this representing a substantial portion of the variance explained, it also shows that a roughly equivalent portion of the variance of the effect of *principled* motivational inferences on trait ratings (i.e. 45.2%) is *not* explained *reputation-signaling* motivational inferences.

## **Supplementary Table 12.** Regression results implied by alternative mediation model specifications of the effect of observability on trait ratings via motivational inferences.

| Dependent variables: | Principled | Reputation-signaling | Principled | Reputation-signaling | Trait ratings |
| --- | --- | --- | --- | --- | --- |
| Predictor variables: | Model 1 | Model 2 | Model 3 | Model 4 | Model 5 |
| Observability | -0.558*** | 0.672*** | -0.252*** | 0.465*** | -0.001 |
| (Public=1) | (0.0302) | (0.0272) | (0.0295) | (0.0258) | (0.0191) |
| Reputation-signaling |  |  | -0.456*** |  | -0.051*** |
|  |  |  | (0.0158) |  | (0.0112) |
| Principled |  |  |  | -0.370*** | 0.575*** |
|  |  |  |  | (0.0129) | (0.0101) |
| Constant | -0.202*** | 0.0294 | -0.188*** | -0.0454 | 0.154*** |
|  | (0.0509) | (0.0459) | (0.0464) | (0.0419) | (0.0300) |
| Control for Experiment | YES | YES | YES | YES | YES |
| Observations | 4,087 | 4,087 | 4,087 | 4,087 | 4,087 |
| R-squared | 0.105 | 0.261 | 0.256 | 0.386 | 0.5481 |

Standard errors in parentheses

*** p<0.01, ** p<0.05, * p<0.1

It could also be the case that we mis-specified the alternative model, and that instead, the effect of *reputation-signaling* motivational inferences on trait ratings are explained by *principled* motivational inferences (i.e. swapping the order of the paths of these mediators in Supplementary Figure 3b). To investigate this possibility, we construct a generalized structural equation model and observe that it provides identical indices of model fit to our first alternative model specification (i.e. AIC: 38,609; BIC: 38,880). Again, turning to the regression results implied by this model (Supplementary Table 12; Columns 1, 4, and 5), we observe that *principled* motivational inferences mediate 30.8% of the effect of *reputation-signaling* motivational inferences on trait ratings. As with our first alternative model, this represents a substantial portion of the variance explained, but also shows that a substantial portion of the variance unexplained (i.e. 69.2%).

Taken together, we believe that the results of these alternatively specified mediation models are inconclusive in providing parsimonious accounts of the mediating effect of observability on trait ratings through motivational inferences. We therefore retain the model we specify in Analysis 2 for three reasons: 1) indices of model fit are slightly better for this model compared to the alternatives we investigate here; 2) neither alternative model is obviously preferable to the other; 3) the model we specify in Analysis 2 is conceptually simpler.

# **Section 8. Complete experimental instructions**

*NOTE: Long solid grey lines represent breaks between pages as experienced by subjects. Long dashed grey lines represent breaks between questions on the same page. Headers for each section (not part of the experiment) displayed in italics.*

## ***Common elements across all experiments***

***Consent***

You are being asked to participate in a research study titled “Social Judgment and Decision- Making”. You were selected to participate in this project because you are an adult over age 18. This study is sponsored by Boston College and the National Science Foundation.

The purpose of this study is social decision-making, and specifically how people judge the decisions and values of others.

This study will be conducted through this online survey. There are no direct benefits to you, but you may feel gratified knowing that you helped further the scholarly work in this research area. There are no costs to you associated with your participation.

This Principal Investigator will exert all reasonable efforts to keep your responses and your identity confidential. **We may have access to, and may maintain in our data collection, your worker ID or user ID for the internet survey platform that you use.  However, aside from your worker ID or user ID, we will not maintain within our research data any information that uniquely identifies you, such as your name, location, or Internet Protocol (IP) address.** In any report we publish, we will not include any information that will make it possible to identify a participant.

Data collected from the experiment will be coded to remove your name or any other personal identifiers. All records will be secured in a locked cabinet in our lab. Access to the records will be limited to the researchers; however, please note that regulatory agencies and the Institutional Review Board and internal Boston College auditors may review the research records. Please note that regulatory agencies, the Boston College Institutional Review Board, and Boston College internal auditors may review research records. **Please also note the organization that operates your Internet survey platform may retain your responses and, additionally, may maintain a link identifying you as the source of those responses.  Your user agreement with the survey platform organization may address this topic.**
  
Your participation is voluntary. If you choose not to participate it will not affect your relations with Boston College. **Some questions on the survey, such as comprehension questions, may be required in order to complete the survey and receive compensation. However, you may still choose to end your participation in the study at any time.**

If you have questions or concerns concerning this research you may contact the Principal Investigator at 617-552-0240 or liane.young@bc.edu. If you have questions about your rights as a research participant, you may contact the Office for Research Protections, Boston College, at 617-552-4778 or [irb@bc.edu](mailto:irb@bc.edu).

If you agree to the statements above and agree to participate in this study, please press the “Consent Given” button below.

- Consent given
- Consent not given

***ID screener and transcription task***

**To begin, please enter your Amazon Mechanical Turk Worker ID here:** 
 
(Please see below for where you can find your Worker ID.) 
 
Your Worker ID starts with the letter A and has 12-14 letters or numbers. It is NOT your email address. If we do not have your correct Worker ID we will not be able to pay you.

________________________________________________________________

Note that your Worker ID can be found on your dashboard page:
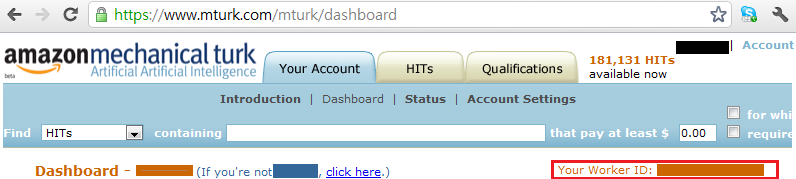


To begin, please type the following paragraph into the box below.


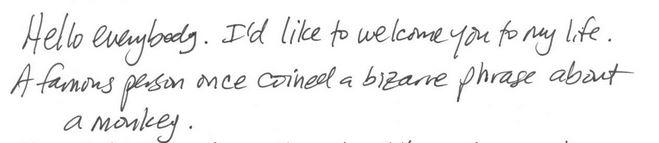


________________________________________________________________

________________________________________________________________

________________________________________________________________

________________________________________________________________

________________________________________________________________

On the following pages, we will describe an individual and ask you some questions about them.

*Demographics*

*NOTE: These questions were administered at the end of all experiments except SI Study 1.*

Gender:

- Male
- Female
- Other/prefer not to say

Age:

________________________________________________________________

Please specify your race. *(Choose one or more categories)*

- White/Caucasian (Anglo/Euro) American
- Black or African American
- Asian or Asian American
- American Indian or Alaska Native
- Native Hawaiian or other Pacific Islander
- Hispanic/Latino
- Multicultural

Highest level of education completed:

- Less than a high school degree
- High School Diploma
- Vocational Training
- Attended College
- Bachelor’s Degree
- Graduate Degree
- Unknown

Please choose the category that describes the total amount of income you earned in [previous year]. Consider all forms of income, including salaries, tips, interest and dividend payments, scholarship support, student loans, parental support, social security, alimony, and child support, and others.

- Under $5,000
- $5,000-$10,000
- $10,001-$15,000
- $15,001-$25,000
- $25,001-$35,000
- $35,001-$50,000
- $50,001-$65,000
- $65,001-$80,000
- $80,001-$100,000
- Over $100,000

Which US political party do you identify with more strongly?

- 1-Strongly Republican
- 2
- 3
- 4-Neutral
- 5
- 6
- 7-Strongly Democrat

What do you think this study is about?

________________________________________________________________

*Attention check – administered in Experiments 10-14*

What mode of transportation do you usually commute with?
*Please select "scooter"*

- Car
- Walk
- Bike
- Scooter
- Public transportation

## ***Manipulations***

***Experiment 1***

*(Condition: Generous, Public)*

Imagine you have a friend named Jen. Jen is always thinking about how her actions will be perceived by others, more than most people do. In particular, she really wants her friends to think that she is a generous person. Jen thinks being generous means giving more of her money or time than is strictly necessary or expected. She is especially generous when others are watching her act since she knows that her reputation for being generous will improve.

*(Condition: Generous, Private)*

Imagine you have a friend named Liz. Liz tries to act in ways that align with her values, regardless of how her actions will be perceived by others. In particular, she thinks it is important to be generous. Liz thinks being generous means giving more of her money or time than is strictly necessary or expected. Though she is generous when she is with others, she is especially generous when no one is watching since she knows that acting in this way is consistent with her values.

*(Condition: Impartial, Public)*

Imagine you have a friend named Jess. Jess is always thinking about how her actions will be perceived by others, more than most people do. In particular, she really wants her friends to think that she is an impartial person. Jess thinks being impartial means treating everyone equally and fairly, without bias. She is especially impartial when others are watching her act since she knows that her reputation for being impartial will improve.

*(Condition: Impartial, Private)*

Imagine you have a friend named Emily. Emily tries to act in ways that align with her values, regardless of how her actions will be perceived by others. In particular, she thinks it is important to be impartial. Emily thinks being impartial means treating everyone equally and fairly, without bias. Though she is impartial when she is with others, she is especially impartial when no one is watching since she knows that acting in this way is consistent with her values.

***Experiments 2 and 3***

*(Condition: Generous, Public)*

Imagine you have a friend named Jen. She really wants her friends to think that she is a generous person. Jen thinks being generous means giving more of her money or time than is strictly necessary or expected. She is especially generous when others are watching her act since she knows that her reputation for being generous will improve.

*(Condition: Generous, Private)*

Imagine you have a friend named Liz. Liz tries to act in ways that align with her values, regardless of how her actions will be perceived by others. In particular, she thinks it is important to be generous. Liz thinks being generous means giving more of her money or time than is strictly necessary or expected. Though she is generous when she is with others, she is even generous when no one is watching since she knows that acting in this way is consistent with her values.

*(Condition: Impartial, Public)*

Imagine you have a friend named Jess. She really wants her friends to think that she is an impartial person. Jess thinks being impartial means treating everyone equally and fairly, without bias. She is especially impartial when others are watching her act since she knows that her reputation for being impartial will improve.

*(Condition: Impartial, Private)*

Imagine you have a friend named Emily. Emily tries to act in ways that align with her values, regardless of how her actions will be perceived by others. In particular, she thinks it is important to be impartial. Emily thinks being impartial means treating everyone equally and fairly, without bias. Though she is impartial when she is with others, she is even impartial when no one is watching since she knows that acting in this way is consistent with her values.

On the next few pages, we will ask you to evaluate Emily's stable personality traits. We'd like to know how you think Emily tends to be *in general*. You should make this judgment however you think is best, but you might consider something like whether or not you could say "She's a very ________ person." The key to these particular judgments is the 'in general.'

***Experiments 4, 5, and 6***

*NOTE: Bracketed sections were displayed in Experiment 4 but not in Experiments 5 and 6.*

*(Condition: Generous, Public)*

Some people think that generosity is a virtue. Generosity usually means giving more of one's money or time than is strictly necessary or expected. Some examples of generosity include:

- Volunteering at a homeless shelter
- Donating money to charities like Doctors without Borders (one of their functions is to provide relief to victims of natural disasters)
- Donating blood during a blood drive (e.g. to the American Red Cross)

Imagine you have a friend named Jen. She really wants her friends to think that she is a generous person. She is especially generous when others are watching her act [since she knows that her reputation for being generous will improve].

*(Condition: Generous, Private)*

Some people think that generosity is a virtue. Generosity usually means giving more of one's money or time than is strictly necessary or expected. Some examples of generosity include:

- Volunteering at a homeless shelter
- Donating money to charities like the Red Cross (one of their functions is to provide relief to victims of natural disasters)
- A teacher staying after school (unpaid) to mentor students

Imagine you have a friend named Liz. Liz tries to act in ways that align with her values, regardless of how her actions will be perceived by others. In particular, she thinks it is important to be generous. Though she is generous when she is with others, she is even generous when no one is watching [since she knows that acting in this way is consistent with her values].

*(Condition: Impartial, Public)*

Some people think that impartiality is a virtue. Impartiality usually means treating everyone equally and fairly, without bias. Some examples of impartiality include:

- Making sure everyone at a social gathering receives the same amount of food (e.g. when four people share a large pizza with eight slices, ensuring everyone gets two)
- Dividing work evenly among all participants in a group project (i.e. not giving your friend less work because you like them)
- Making auditions or job applications blind (i.e. evaluators can't see applicants' faces) so that subtle, unconscious biases against particular genders or ethnicities don't enter into the decision-making process

Imagine you have a friend named Jess. She really wants her friends to think that she is an impartial person. She is especially impartial when others are watching her act [since she knows that her reputation for being impartial will improve].

*(Condition: Impartial, Private)*

Some people think that impartiality is a virtue. Impartiality usually means treating everyone equally and fairly, without bias. Some examples of impartiality include:

- Making sure everyone at a social gathering receives the same amount of food (e.g. when four people share a large pizza with eight slices, ensuring everyone gets two)
- Dividing work evenly among all participants in a group project (i.e. not giving your friend less work because you like them)
- Making auditions or job applications blind (i.e. evaluators can't see applicants' faces) so that subtle, unconscious biases against particular genders or ethnicities don't enter into the decision-making process

Imagine you have a friend named Emily. Emily tries to act in ways that align with her values, regardless of how her actions will be perceived by others. In particular, she thinks it is important to be impartial. Though she is impartial when she is with others, she is even impartial when no one is watching [since she knows that acting in this way is consistent with her values].

On the next few pages, we will ask you to evaluate Emily's stable personality traits. We'd like to know how you think Emily tends to be *in general*. You should make this judgment however you think is best, but you might consider something like whether or not you could say "She's a very ________ person." The key to these particular judgments is the 'in general.'

***Experiments 7, 8, and 9***

*(Condition: Generous, Public)*

Some people think that generosity is a virtue. Generosity usually means giving an abundance of one's money or time.

Imagine you know someone named Jen who bought a friend an expensive gift, gave a waiter a large tip, and stayed late to help a coworker. She did these things in public; therefore, other people knew that she did them.

*(Condition: Generous, Private)*

Some people think that generosity is a virtue. Generosity usually means giving an abundance of one's money or time.

Imagine you know someone named Liz who bought a friend an expensive gift, gave a waiter a large tip, and stayed late to help a coworker. She did these things in private; therefore, other people did not know that she did them.

*(Condition: Impartial, Public)*

Some people think that impartiality is a virtue. Impartiality usually means treating everyone equally and fairly, without bias.

Imagine you know someone named Jess who gave her children equal allowances, conducted a blind audition, and drew names from a hat for a project at work. She did these things in public; therefore, other people knew that she did them.

*(Condition: Impartial, Private)*

Some people think that impartiality is a virtue. Impartiality usually means treating everyone equally and fairly, without bias.

Imagine you know someone named Emily who gave her children equal allowances, conducted a blind audition, and drew names from a hat for a project at work. She did these things in private; therefore, other people did not know that she did them.

***Experiments 7 and 8***

*(Condition: Generous, Baseline)*

Some people think that generosity is a virtue. Generosity usually means giving an abundance of one's money or time.

Imagine you know someone named Lisa who buys a friend an expensive gift, gives a waiter a large tip, and stays late to help a coworker.

*(Condition: Impartial, Baseline)*

Some people think that impartiality is a virtue. Impartiality usually means treating everyone equally and fairly, without bias.

***Experiment 10***

Some people think that generosity is a virtue. Generosity usually means giving an abundance of one's money or time.

Imagine you know someone named [name] who giving someone a hand carrying groceries, shared food with friends, and gave someone praise.

*(Condition: Public)*

She did these things in public; therefore, other people knew that she did them.

*(Condition: Private)*

She did these things in private; therefore, other people did not know that she did them.

*(Condition: Reputation-signaling motivation)*

She was motivated to do these things for the following reasons: she wants others to think that she is generous, and she thinks she will benefit from others perceiving her as generous.

She was NOT motivated to do these things for the following reasons: she wants to be generous, she wants to benefit others, she thought it was the right thing to do, she wants others to be generous and she was trying to lead by example.

*(Condition: Principled motivation)*

She was motivated to do these things for the following reasons: she wants to be generous, she wants to benefit others, she thought it was the right thing to do, she wants others to be generous and she was trying to lead by example.

She was NOT motivated to do these things for the following reasons: she wants others to think that she is generous, and she thinks she will benefit from others perceiving her as generous.

***Experiment 11***

*(Condition: Generous, Public)*

Some people think that generosity is a virtue. Generosity usually means giving an abundance of one's money or time.

Imagine you know someone named Jen who gave someone a hand carrying groceries, shared food with friends, and gave someone praise. She did these things in public; therefore, other people knew that she did them.

*(Condition: Generous, Private)*

Some people think that generosity is a virtue. Generosity usually means giving an abundance of one's money or time.

Imagine you know someone named Liz who gave someone a hand carrying groceries, shared food with friends, and gave someone praise. She did these things in private; therefore, other people did not know that she did them.

*(Condition: Impartial, Public)*

Some people think that impartiality is a virtue. Impartiality usually means treating everyone equally and fairly, without bias.

Imagine you know someone named Jess who stayed out of an argument, divided food by cutting and letting the other person pick which piece they want, and helped to moderate when your friends had a disagreement. She did these things in public; therefore, other people knew that she did them.

*(Condition: Impartial, Private)*

Some people think that impartiality is a virtue. Impartiality usually means treating everyone equally and fairly, without bias.

Imagine you know someone named Emily who stayed out of an argument, divided food by cutting and letting the other person pick which piece they want, and helped to moderate when your friends had a disagreement. She did these things in private; therefore, other people did not know that she did them.

***Experiment 12***

*(Condition: Generous, Public)*

We will present you with a few scenarios and ask you some questions about them.

All of the scenarios have to do with the idea of “generosity”, which some people think is a virtue.

Generosity usually means giving an abundance of one's money or time.

-NEW SCENARIO-

[NOTE: The next set of questions will be based on the following scenario; we will include this text with the questions that follow.]

Imagine you know someone named Jen who gave someone a hand carrying groceries. She did this in public; therefore, other people knew that she did it.

-NEW SCENARIO-

[NOTE: The next set of questions will be based on the following scenario; we will include this text with the questions that follow.]

Imagine you know someone named Emily who shared food with friends. She did this in public; therefore, other people knew that she did it.

-NEW SCENARIO-

[NOTE: The next set of questions will be based on the following scenario; we will include this text with the questions that follow.]

Imagine you know someone named Liz who gave someone praise. She did this in public; therefore, other people knew that she did it.

*(Condition: Generous, Private)*

We will present you with a few scenarios and ask you some questions about them.

All of the scenarios have to do with the idea of “generosity”, which some people think is a virtue.

Generosity usually means giving an abundance of one's money or time.

-NEW SCENARIO-

[NOTE: The next set of questions will be based on the following scenario; we will include this text with the questions that follow.]

Imagine you know someone named Jen who gave someone a hand carrying groceries. She did this in private; therefore, other people did not know that she did it.

-NEW SCENARIO-

[NOTE: The next set of questions will be based on the following scenario; we will include this text with the questions that follow.]

Imagine you know someone named Emily who shared food with friends. She did this in private; therefore, other people did not know that she did it.

-NEW SCENARIO-

[NOTE: The next set of questions will be based on the following scenario; we will include this text with the questions that follow.]

Imagine you know someone named Liz who gave someone praise. She did this in private; therefore, other people did not know that she did it.

*(Condition: Impartial, Public)*

We will present you with a few scenarios and ask you some questions about them.

All of the scenarios have to do with the idea of “impartiality”, which some people think is a virtue.

Impartiality usually means treating everyone equally and fairly, without bias.

-NEW SCENARIO-

[NOTE: The next set of questions will be based on the following scenario; we will include this text with the questions that follow.]

Imagine you know someone named Jen who stayed out of an argument. She did this in public; therefore, other people knew that she did it.

-NEW SCENARIO-

[NOTE: The next set of questions will be based on the following scenario; we will include this text with the questions that follow.]

Imagine you know someone named Emily who divided food by cutting and letting the other person pick which piece they want. She did this in public; therefore, other people knew that she did it.

-NEW SCENARIO-

[NOTE: The next set of questions will be based on the following scenario; we will include this text with the questions that follow.]

Imagine you know someone named Liz who helped to moderate when your friends had a disagreement. She did this in public; therefore, other people knew that she did it.

*(Condition: Impartial, Private)*

We will present you with a few scenarios and ask you some questions about them.

All of the scenarios have to do with the idea of “impartiality”, which some people think is a virtue.

Impartiality usually means treating everyone equally and fairly, without bias.

-NEW SCENARIO-

[NOTE: The next set of questions will be based on the following scenario; we will include this text with the questions that follow.]

Imagine you know someone named Jen who stayed out of an argument. She did this in private; therefore, other people did not know that she did it.

-NEW SCENARIO-

[NOTE: The next set of questions will be based on the following scenario; we will include this text with the questions that follow.]

Imagine you know someone named Emily who divided food by cutting and letting the other person pick which piece they want. She did this in private; therefore, other people did not know that she did it.

-NEW SCENARIO-

[NOTE: The next set of questions will be based on the following scenario; we will include this text with the questions that follow.]

Imagine you know someone named Liz who helped to moderate when your friends had a disagreement. She did this in private; therefore, other people did not know that she did it.

***Experiment 13***

*(Condition: Generous, Public)*

Some people think that generosity is a virtue. Generosity usually means giving an abundance of one's money or time.

Imagine you know someone named Jen who bought someone a meal, donated blood, and volunteered at an animal shelter. She did these things in public; therefore, other people knew that she did them.

*(Condition: Generous, Private)*

Some people think that generosity is a virtue. Generosity usually means giving an abundance of one's money or time.

Imagine you know someone named Liz who bought someone a meal, donated blood, and volunteered at an animal shelter. She did these things in private; therefore, other people did not know that she did them.

*(Condition: Impartial, Public)*

Some people think that impartiality is a virtue. Impartiality usually means treating everyone equally and fairly, without bias.

Imagine you know someone named Jess who divided food by cutting it and letting the other person pick which piece they wanted, learned to pronounce others' names regardless of their country of origin, and listened to both parties in a conflict equally. She did these things in public; therefore, other people knew that she did them.

*(Condition: Impartial, Private)*

Some people think that impartiality is a virtue. Impartiality usually means treating everyone equally and fairly, without bias.

Imagine you know someone named Emily who divided food by cutting it and letting the other person pick which piece they wanted, learned to pronounce others' names regardless of their country of origin, and listened to both parties in a conflict equally. She did these things in private; therefore, other people did not know that she did them.

***Experiment 14***

*(Condition: Generosity)*

Some people think that generosity is a virtue. Generosity usually means giving an abundance of one's money or time.

Imagine you know someone named Jen who bought someone a meal, donated blood, and volunteered at an animal shelter.

*(Condition: Impartiality)*

Some people think that impartiality is a virtue. Impartiality usually means treating everyone equally and fairly, without bias.

Imagine you know someone named Jess who divided food by cutting it and letting the other person pick which piece they wanted, learned to pronounce others' names regardless of their country of origin, and listened to both parties in a conflict equally.

*(Condition: Public)*

She did these things in public; therefore, other people knew that she did them.

*(Condition: Private)*

She did these things in private; therefore, other people did not know that she did them.

*(Condition: Reputation-signaling motivation)*

She was motivated to do these things for the following reasons: she wants others to think that she is generous, and she thinks she will benefit from others perceiving her as generous.

She was NOT motivated to do these things for the following reasons: she wants to be generous, she wants to benefit others, she thought it was the right thing to do, she wants others to be generous and she was trying to lead by example.

*(Condition: Principled motivation)*

She was motivated to do these things for the following reasons: she wants to be generous, she wants to benefit others, she thought it was the right thing to do, she wants others to be generous and she was trying to lead by example.

She was NOT motivated to do these things for the following reasons: she wants others to think that she is generous, and she thinks she will benefit from others perceiving her as generous.

## ***Dependent measures***

*NOTE: All sliders recorded values from 0-100 but did not display these values to participants.*

***All experiments***

*Moral goodness rating*

How morally good is [name]?

|  | Extremely morally bad | Neither morally good nor morally bad | Extremely morally good |
| --- | --- | --- | --- |

|  | 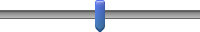 |
| --- | --- |

*Trait rating*

How [generous/impartial] is [name]?

|  | Extremely selfish | Neither generous nor selfish | Extremely generous |
| --- | --- | --- | --- |

|  | 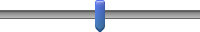 |
| --- | --- |

*Self-benefit*

***Experiments 2 and 3:***

How much do you think [name] will personally benefit from being perceived as generous by others?

|  | Not at all | Very much |
| --- | --- | --- |

|  | 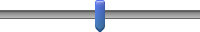 |
| --- | --- |

***Experiments 6-9, 11-13:***

How much do you think [name] is motivated to act [generously/impartially] **because she thinks she will personally benefit from acting this way**?

|  | Not at all | Very much |
| --- | --- | --- |

|  | 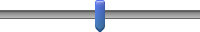 |
| --- | --- |

*Other-benefit*

***Experiments 4 and 5:***

How much do you think another person would benefit from interacting with [name]?

|  | Not at all | Very much |
| --- | --- | --- |

|  | 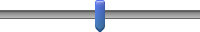 |
| --- | --- |

***Experiments 6-9, 11-13:***

How much do you think [name] is motivated to act [generously/impartially] **because she wants to benefit others**?

|  | Not at all | Very much |
| --- | --- | --- |

|  | 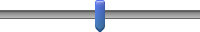 |
| --- | --- |

*Self-presentation*

***Experiments 4 and 5:***

Think about [name]’s motivation for acting the way she does. How much do you think [name] is acting this way because...

|  | 1: Not at all | 2 | 3 | 4 | 5 | 6 | 7: Very much |
| --- | --- | --- | --- | --- | --- | --- | --- |
| ...she is thinking about what others would think of her |  |  |  |  |  |  |  |
| ...it's important to her that others positively evaluate her |  |  |  |  |  |  |  |
| ...it's important to her that others accept her |  |  |  |  |  |  |  |

***Experiments 6-9, 11-13:***

How much do you think [name] is motivated to act [generously/impartially] **because she is trying to make others think she is [generous/impartial]**?

|  | Not at all | Very much |
| --- | --- | --- |

|  | 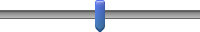 |
| --- | --- |

*Virtue identification*

***Experiments 4 and 5:***

Think about [name]’s motivation for acting the way she does. How much do you think [name] is acting this way because...

|  | 1: Not at all | 2 | 3 | 4 | 5 | 6 | 7: Very much |
| --- | --- | --- | --- | --- | --- | --- | --- |
| ...she thinks it is important to act in this way |  |  |  |  |  |  |  |
| ...she likes acting this way |  |  |  |  |  |  |  |
| ...she values doing so |  |  |  |  |  |  |  |

***Experiments 6-9, 11-13:***

How much do you think [name] is motivated to act [generously/impartially] **because she wants to be [generous/impartial]**?

|  | Not at all | Very much |
| --- | --- | --- |

|  | 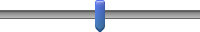 |
| --- | --- |

*Norm-signaling motivation*

***Experiments 4 and 5:***

Think about [name]’s motivation for acting the way she does. How much do you think [name] is acting this way because...

|  | 1: Not at all | 2 | 3 | 4 | 5 | 6 | 7: Very much |
| --- | --- | --- | --- | --- | --- | --- | --- |
| ...she is modelling the behavior she wants others to engage in |  |  |  |  |  |  |  |
| ...she wants others to follow her example |  |  |  |  |  |  |  |
| ...it's important to her to show others how she thinks everyone should behave |  |  |  |  |  |  |  |

***Experiments 6-9, 11-13:***

How much do you think [name] is motivated to act [generously/impartially] **because she wants others to be [generous/impartial], and she is trying to lead by example**?

|  | Not at all | Very much |
| --- | --- | --- |

|  | 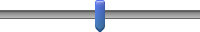 |
| --- | --- |

*Moral rule*

***Experiments 6-9, 11-13:***

How much do you think [name] is motivated to act [generously/impartially] **because she thinks it is the right thing to do**?

|  | Not at all | Very much |
| --- | --- | --- |

|  | 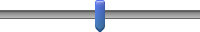 |
| --- | --- |

*2^nd^-party benefit*

***Experiments 2 and 3:***

How much do you think you would benefit from interacting with [name]?

|  | Not at all | The same as with anyone else | Very much |
| --- | --- | --- | --- |

|  | 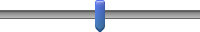 |
| --- | --- |

1. Kraft-Todd, G., Kleiman-Weiner, M., & Young, L. (2022). Operationalizing and Dissociating Virtues from the ‘Bottom up’: A Case study of Generosity vs. Impartiality. https://doi.org/10.31234/osf.io/3paqs [↑](#footnote-ref-1)
2. E.g. Newman, G. E., & Cain, D. M. (2014). Tainted altruism: When doing some good is evaluated as worse than doing no good at all. *Psychological science*, *25*(3), 648-655. [↑](#footnote-ref-2)
3. Hayes, A. F. (2013). Introduction to mediation, moderation, and conditional process analysis: A regression-based approach. Guilford Press. [↑](#footnote-ref-3)
